# Screen Time, Unhealthy Eating Behaviors, and Associated Health Risks in Children: A Narrative Review

**DOI:** 10.3390/children13070887

**Published:** 2026-06-30

**Authors:** Valeria Calcaterra, Hellas Cena, Luca Marin, Caterina Cavallo, Silvia Taranto, Maria Vittoria Conti, Pamela Patanè, Luca Guardamagna, Ester Minardi, Lea Bellingeri, Dario Silvestri, Matteo Vandoni, Gianvincenzo Zuccotti

**Affiliations:** 1 Department of Internal Medicine and Therapeutics, University of Pavia, 27100 Pavia, Italy; 2 Pediatric Department, Buzzi Children’s Hospital, 20154 Milano, Italy; silvia.taranto@unimi.it (S.T.); lea.bellingeri@unimi.it (L.B.); gianvincenzo.zuccotti@unimi.it (G.Z.); 3 Laboratory of Dietetics and Clinical Nutrition, Department of Public Health, Experimental and Forensic Medicine, University of Pavia, 27100 Pavia, Italy; hellas.cena@unipv.it (H.C.); mariavittoria.conti@unipv.it (M.V.C.); 4 Clinical Nutrition and Dietetics Unit, ICS Maugeri IRCCS, 27100 Pavia, Italy; 5 Laboratory of Adapted Motor Activity (LAMA), Department of Public Health, Experimental Medicine and Forensic Science, University of Pavia, 27100 Pavia, Italy; luca.marin@unipv.it (L.M.); caterina.cavallo01@universitadipavia.it (C.C.); ester.minardi01@universitadipavia.it (E.M.); matteo.vandoni@unipv.it (M.V.); 6 Laboratory for Rehabilitation and Orthopedic Surgery (LAROS), Department of Clinical, Diagnostic and Pediatric Sciences, University of Pavia, 27100 Pavia, Italy; pamela.patane@unipv.it (P.P.); luca.guardamagna@grupposandonato.it (L.G.); 7 Asomi College of Sciences, School of Medicine, 1014 Pembroke, Malta; dario.silvestri@acs-college.com; 8 Department of Biomedical and Clinical Science, University of Milano, 20157 Milano, Italy

**Keywords:** screen time, eating behaviors, childhood obesity, digital food marketing, cardiometabolic risk

## Abstract

**Highlights:**

**What are the main findings?**

- Screen exposure in children and adolescents is associated with unhealthy eating behaviors, including mindless eating, frequent snacking, breakfast skipping, irregular meals, and higher consumption of ultra-processed, energy-dense foods.
- These behaviors are associated with multiple pathways, including digital food marketing, sedentary behavior, sleep and circadian disruption, appetite-related hormonal changes, neurocognitive mechanisms, cardiometabolic risk, and mental health vulnerability.

**What are the implications of the main finding?**

- Prevention should address not only screen duration, but also screen content, timing, context, family routines, sleep hygiene, physical activity, and digital/media literacy.
- Multilevel strategies involving families, schools, healthcare professionals, communities, and public health policies are needed to address screen-related dietary, metabolic, and psychological correlates and potential risks in pediatric populations.

**Abstract:**

**Introduction:** The widespread use of digital devices has significantly increased screen time among children and adolescents, raising concerns about its effects on dietary behaviors and health outcomes. This narrative review aimed to examine the relationship between screen exposure, unhealthy eating behaviors, and associated physical and mental health risks in pediatric populations. **Methods:** A narrative review of the literature was conducted using PubMed, Scopus, and Web of Science databases, with the final search updated on 30 April 2026. Studies involving individuals aged 0–18 years were included if they investigated screen time, dietary behaviors, obesity, cardiometabolic risk, sleep, or mental health outcomes. Observational studies, interventional studies, systematic reviews, and meta-analyses were considered. Findings were synthesized qualitatively according to pre-specified thematic domains, with greater interpretive weight given to systematic reviews, meta-analyses, randomized trials, longitudinal cohorts, and large population-based studies. **Results:** Excessive screen time was consistently associated with unhealthy eating behaviors, including increased snacking, mindless eating, breakfast skipping, and higher consumption of ultra-processed and energy-dense foods. Digital food marketing and exposure to food advertising may influence children’s food preferences and intake. High screen exposure was also associated with sedentary behavior, sleep disturbances, circadian dysregulation, and altered appetite-related hormones such as leptin and ghrelin. These pathways may contribute to, or be consistent with, obesity, insulin resistance, dyslipidemia, hypertension, and metabolic syndrome, although most evidence remains observational. Moreover, excessive screen use was associated with anxiety, depression, emotional eating, binge-eating behaviors, and body image dissatisfaction, particularly among adolescents. **Conclusions**: Screen time may represent a modifiable behavioral and environmental correlate of dietary habits, metabolic health, and psychological wellbeing in children and adolescents. Prevention strategies should focus not only on reducing screen duration but also on improving the quality and context of media use through family-based interventions, school programs, digital literacy, promotion of physical activity, and regulation of digital food marketing. Further longitudinal and interventional studies are needed to clarify causal relationships and develop effective prevention strategies.

## 1. Introduction

In recent decades, the widespread availability of digital devices has profoundly transformed children’s daily lives [1], leading to a substantial increase in screen time from early childhood [2,3]. Television, once the primary source of screen exposure, has progressively been complemented, and in many cases replaced, by smartphones, tablets, video games, and social media platforms [4]. As a result, children are now exposed to multiple forms of digital media for extended periods throughout the day, often exceeding recommended limits [2,3].

This rise in screen time has raised growing concerns within the pediatric and public health communities, particularly due to its potential impact on lifestyle behaviors [1]. Among these, unhealthy eating behaviors have emerged as a key area of interest. Evidence suggests that prolonged screen exposure is associated with increased consumption of energy-dense, nutrient-poor foods, irregular eating patterns, and a higher frequency of snacking [5,6,7]. These behaviors may be driven by several factors, including distracted eating, exposure to food advertising, and the influence of digital environments on food preferences and choices [8,9].

Importantly, these behavioral changes are occurring in parallel with the increasing prevalence of childhood overweight and obesity, which represent major global health challenges [10]. Early-life dietary habits and sedentary behaviors are known to track into adulthood, contributing to long-term cardiometabolic risks such as insulin resistance, dyslipidemia, and hypertension [11,12]. Moreover, emerging evidence highlights potential links between screen use, eating behaviors, and mental health outcomes, including emotional eating and body image concerns.

Understanding the complex interplay between screen time, dietary behaviors, and health outcomes is therefore essential for developing effective prevention strategies. Multiple biological and behavioral mechanisms have been proposed, including reduced energy expenditure due to sedentary behavior, neurocognitive responses to food-related stimuli, and disruptions in sleep and circadian rhythms that may affect appetite regulation [13,14,15].

In this context, the aim of the present narrative review is to synthesize current evidence on the association between screen time and unhealthy eating behaviors in children, and to explore the underlying mechanisms and related health risks. By providing an integrated overview of these interrelated factors, this review seeks to inform clinical practice and public health interventions targeting pediatric populations.

## 2. Methods

This study was conducted as a narrative review aimed at summarizing and critically appraising the literature on the relationship between screen exposure, unhealthy eating behaviors, and associated health-related outcomes in children and adolescents. The search was performed in PubMed, Scopus, and Web of Science and was last updated on 30 April 2026. The review followed a transparent narrative approach: screen exposure is used as the overarching construct encompassing duration, device type, content, timing, context, and purpose of use; screen time refers specifically to the duration of screen-based activity; and screen use refers to behavioral or contextual patterns, which are qualified where appropriate (e.g., recreational, evening, or mealtime screen use). Although no formal PRISMA flow diagram or quantitative meta-analysis was planned, the search strategy, eligibility criteria, evidence prioritization, and main limitations of the review process were defined a priori.

The search combined controlled vocabulary and free-text terms related to screen exposure, pediatric populations, eating behavior, and health outcomes. The core PubMed strategy was: (“screen time” OR “screen exposure” OR “digital media” OR television OR smartphone OR tablet OR videogame* OR “social media” OR “digital marketing”) AND (child* OR adolescen* OR pediatric OR preschool OR youth) AND (“eating behavior” OR “dietary habits” OR “food intake” OR snacking OR “breakfast skipping” OR “ultra-processed food” OR “food advertising” OR “food marketing”) AND (obesity OR adiposity OR “body mass index” OR cardiometabolic OR “insulin resistance” OR dyslipidemia OR hypertension OR sleep OR depression OR anxiety OR “emotional eating” OR “binge eating” OR “body image”). Equivalent terms were adapted for Scopus and Web of Science (Table S1). Filters were limited to English-language articles involving humans aged 0–18 years; when studies included mixed-age samples, they were considered only if pediatric or adolescent data were reported separately or were directly relevant to the developmental interpretation of findings.

Eligible publications included observational studies, longitudinal cohort studies, randomized or quasi-experimental interventions, systematic reviews, meta-analyses, guideline documents, and policy-relevant reports. Studies were excluded if they focused exclusively on adults, were case reports or editorials without original synthesis, were non-peer-reviewed sources, or did not address screen exposure in relation to dietary behaviors, metabolic outcomes, sleep, or mental health.

Titles and abstracts were screened for relevance, followed by full-text evaluation of potentially eligible articles. Given the narrative design of the review, study selection was guided by relevance to the research objectives rather than by strict systematic review procedures. Studies were prioritized according to methodological strength, population size, developmental specificity, clarity of exposure and outcome definitions, and recency. Greater weight was given to systematic reviews, meta-analyses, randomized trials, longitudinal cohorts, and large population-based studies, whereas small cross-sectional studies were interpreted with greater caution.

For each included study, information was extracted on study design, country or setting, age range, sample characteristics, type and timing of screen exposure, exposure assessment method, dietary or behavioral outcomes, health outcomes, and main findings. Because of the heterogeneity of devices, exposure metrics, outcome definitions, and study designs, the evidence was synthesized qualitatively rather than quantitatively. The synthesis was organized into the following domains: types and epidemiology of screen exposure, unhealthy eating behaviors, digital food marketing, sedentary and neurocognitive mechanisms, sleep and circadian pathways, obesity and cardiometabolic risk, mental health and disordered eating outcomes, prevention strategies, and research gaps. Although no formal risk-of-bias assessment was performed, methodological limitations and inconsistencies across studies were considered in the interpretation of the evidence.

## 3. Screen Time in Children

### 3.1. Types of Exposure

Screen exposure in childhood now represents a broad and multidimensional construct that includes television, smartphones, tablets, computers, video games, and social media platforms, particularly among older children and adolescents [16,17,18,19]. This reflects both the expansion of digital technologies in the home and a shift from mainly passive, scheduled, and family-shared television viewing toward portable, interactive, personalized, and on-demand media use [17,18,20,21]. Although television remains relevant in early childhood, smartphones and tablets have become increasingly common due to their portability, accessibility, and multiple functions, including video viewing, gaming, communication, and educational applications [17,22,23].

A further conceptual distinction that has become increasingly relevant is that between passive and active forms of exposure. Passive exposure includes traditional television viewing and background television, whereas active exposure includes gaming, app use, and other forms of interactive engagement with digital content [17,24]. This distinction matters because the developmental correlates of screen use appear to vary according to the type of activity performed, the level of interactivity involved, and the social circumstances in which exposure occurs. For example, video games and computer-based activities may involve higher levels of cognitive and behavioral engagement than passive television viewing, while social media introduces additional dimensions related to peer interaction, identity formation, and social comparison, particularly in adolescence [21,25,26]. At the same time, the literature increasingly emphasizes that screen exposure should not be categorized exclusively by device type, because the same device can be used for very different purposes, including entertainment, education, emotional regulation, or social interaction [18,20,21].

Another important layer of complexity concerns context. Screen use may occur alone, with siblings, or in the presence of caregivers, and this contextual variation appears to influence its developmental significance [18,21,24]. Co-viewing and caregiver companionship may buffer some adverse associations by promoting language input, joint attention, and emotional regulation, whereas solitary use or background exposure may reduce opportunities for reciprocal parent–child interaction [16,18,24,27]. In addition, several studies suggest that family ecology, including parental screen habits, household rules, background television, and technology-related interruptions in family interaction, plays a substantial role in shaping children’s media exposure patterns [20,23,28,29]. Taken together, these findings support the view that screen exposure in childhood is not a single, homogeneous construct, but rather a constellation of interrelated media behaviors that vary by device, content, context, and purpose of use.

A conceptual framework for this review is therefore that screen-related health risks are not determined by duration alone. Risk may vary according to device type, content, timing, purpose of use, developmental stage, parental mediation, family rules, mealtime context, evening exposure, and individual vulnerability. This framework is used throughout the review to avoid treating screen exposure as a homogeneous construct and to distinguish recreational, educational, passive, interactive, solitary, shared, daytime, and bedtime screen use.

### 3.2. Epidemiological Trends

Epidemiological evidence consistently indicates that children’s screen exposure has increased over time, with both longer daily duration and earlier onset. The age of first exposure has declined markedly, from approximately 4 years in the 1970s to infancy in recent cohorts [20]. Current data suggest that screen devices are used by a very high proportion of children before the age of four, with average exposure commonly ranging between 1 and 2 h per day in preschool populations and increasing during weekends [16,24,30]. Adherence to recommended limits remains low, with many children exceeding the threshold of ≤2 h per day [16]. Longitudinal and surveillance data further suggest that screen exposure has risen alongside access to mobile devices and digital technologies [20,31].

This pattern has been documented across diverse settings, including Canada, India, Portugal, Spain, Thailand, Turkey, and Jordan, although estimates vary according to age, study design, and the operational definition of screen time [16,22,28,32,33,34]. Overall, the increase appears to reflect both greater device availability and the integration of digital media into daily routines for leisure, education, communication, and family management [17,18,25].

Screen exposure also tends to increase with age. While excessive screen exposure is already common in preschool children, older children and adolescents generally report longer and more diversified media engagement, including gaming, social interaction, and independent device use [22,26,32]. Repeated cross-sectional data from Portugal showed that mobile media added to, rather than replaced, television viewing between 2009/10 and 2016/17 [22]. Similarly, Norwegian device-measured data documented increased sedentary time between 2005 and 2018, consistent with the spread of smartphones, tablets, and near-universal internet access [31]. Review-level evidence further suggests that the COVID-19 period accelerated pre-existing trajectories toward higher screen use [25].

Differences by gender and socioeconomic context are recurrent but not uniform. Boys often report greater engagement in video gaming and recreational screen use, whereas girls may spend more time in other media activities depending on age and setting [26,32]. Socioeconomic gradients are similarly complex: lower parental education, unemployment, and fewer household resources have been associated with excessive screen use in some studies, while greater income may increase device access in others [22,33]. Family-level factors, including parental screen habits, absence of rules, bedroom devices, background television, and technoference, consistently contribute to children’s exposure patterns [23,29]. Overall, screen exposure is increasingly prevalent and socially patterned, and cannot be adequately described by duration alone.

### 3.3. International Recommendations

In response to the expansion of screen exposure in childhood, several professional and public health organizations have issued recommendations aimed at limiting excessive screen use and promoting healthier media habits. The most widely cited include those from the American Academy of Pediatrics (AAP), the World Health Organization (WHO), the Canadian Paediatric Society, and national pediatric bodies such as the Indian Academy of Pediatrics [32,35,36,37,38]. Across these frameworks, there is broad agreement that children under 2 years of age should ideally avoid screen exposure, with video communication generally treated as a limited exception, while children aged 2 to 5 years should have routine and regular screen time restricted to about 1 h per day; in older children and adolescents, recommendations often converge around limiting recreational screen time to less than 2 h per day [32,33,35,36,37,38,39].

At the same time, recent literature suggests that contemporary recommendations are moving beyond purely time-based models toward a more nuanced understanding of “healthy screen use.” Reviews and guideline-oriented papers increasingly stress that the quality of content, the purpose of use, and the social context of media exposure are essential considerations alongside duration [17,18,25]. Educational content, caregiver co-use, and emotionally responsive supervision are often presented as protective elements, whereas solitary, unsupervised, or habitual use for behavioral regulation may be more problematic [18,20,21,24,27].

Despite the availability of these recommendations, adherence is consistently low across many populations. Preschool and school-age children frequently exceed suggested thresholds, and this occurs across countries with different cultural and socioeconomic profiles [22,32,33,34]. This mismatch between recommendations and real-world practice likely reflects the pervasive presence of digital devices, the normalization of media use within family life, and the multiple roles screens now play in entertainment, education, communication, and parental coping [18,25,29]. Therefore, although international guidelines remain essential as a public health reference point, the current evidence suggests that future recommendations may benefit from becoming more context-sensitive and family-centered, integrating considerations of device type, content, co-use, and household media ecology rather than relying exclusively on daily duration thresholds.

Developmental stage is an additional interpretive dimension. Preschool children may be particularly influenced by parental screen habits, background television, caregiver co-use, and mealtime routines [5]; school-aged children may be more affected by household rules, television viewing, gaming, and availability of snacks during screen use [40]; adolescents may be more vulnerable to social media, influencer marketing, peer comparison, emotional regulation difficulties, body image concerns, and greater autonomy over food choices [40]. These developmental differences should be considered when interpreting associations and designing preventive interventions.

## 4. Associated Unhealthy Eating Behaviors

### 4.1. Eating While Watching (Mindless Eating)

Eating while watching screens is one of the most frequently described unhealthy eating behaviors in children and adolescents exposed to prolonged screen time. Meals and snacks consumed during television viewing, gaming, or smartphone use may promote a state of reduced eating awareness, in which attention is diverted from the amount and pace of food intake, sensory experience, and internal hunger and satiety cues toward external digital stimuli. This pattern is commonly described as “mindless eating” [13]. By shifting attention from the eating episode to external digital stimuli, screen use may impair self-regulation of food intake and favor passive overconsumption [41,42,43,44,45].

Several studies suggest that children who eat while watching screens tend to consume larger portion sizes, eat more rapidly, and show a greater preference for highly palatable, energy-dense foods [44,46,47]. Screen-based eating may also reduce the structure and social quality of meals, particularly when it replaces shared family meals or occurs in the absence of caregiver supervision [18,20,23,28,29]. In younger children, parental modeling, household media rules, and family eating routines appear especially relevant, as screen use during meals is often embedded within broader family habits [18,23,24,28]. The importance of family environment and eating routines in shaping children’s dietary behaviors has also been described in adolescent populations.

Furthermore, repeated exposure to food cues during screen-based activities may reinforce hedonic eating mechanisms independently of physiological hunger [41,42,43,44,45]. This pattern may be particularly problematic in children with higher responsiveness to food cues or lower inhibitory control, who may be more vulnerable to overeating in response to external food-related stimuli [41,42,43,44,45,48]. However, as much of the available evidence is observational, these associations should be interpreted cautiously and considered within the broader context of family routines, dietary environment, and overall lifestyle behaviors [49].

### 4.2. Food Advertising and Digital Food Marketing

Food advertising represents one of the main pathways through which screen exposure may influence children’s eating behaviors and food preferences [13,41,50]. Across television, streaming platforms, online games, social media, and influencer-generated content, children and adolescents are frequently exposed to marketing strategies promoting energy-dense, nutrient-poor, and ultra-processed foods, including sugar-sweetened beverages, fast food, salty snacks, and confectionery products [41,42,43,44,45].

Younger children are particularly vulnerable to food advertising because of their limited ability to recognize persuasive intent, whereas adolescents may be more susceptible to peer influence, social comparison, and emotionally driven marketing strategies [22,44,51]. Compared with traditional television advertising, digital food marketing is especially concerning because it is often personalized, immersive, interactive, and embedded within entertainment content, making commercial messages more difficult to identify and critically interpret [22,44,51].

Social media influencers and algorithm-driven advertising may further reinforce unhealthy food norms and normalize frequent consumption of ultra-processed foods, particularly among adolescents who use digital platforms for entertainment, social interaction, and identity formation [44]. Experimental studies suggest that food advertising may acutely increase energy intake even in the absence of hunger, likely through activation of reward-related neural pathways and attentional bias toward palatable foods [41,42,43,44]. These effects may be amplified in children with overweight or obesity, who may display greater responsiveness to food cues [45,48]. In this context, digital food marketing differs from traditional television advertising because it can be personalized, immersive, interactive, algorithmically targeted, and embedded within entertainment content. Commercial messages may appear in short videos, livestreaming, advergames, social media challenges, influencer posts, and platform recommendations, making persuasive intent difficult to recognize. This is particularly relevant for minors, who may have limited advertising literacy, and for adolescents, whose food choices may be influenced by peer norms, identity formation, and appearance-related or emotion-based marketing. Consequently, digital food marketing should be considered a commercial determinant of unhealthy dietary behavior and a priority target for public health regulation [8].

### 4.3. Altered Dietary Quality and Food Choices

Excessive screen exposure has been associated with less healthy dietary patterns across childhood and adolescence [5,6,13,46]. Several observational studies report that children with higher daily screen time consume greater amounts of sugar-sweetened beverages, fast food, salty snacks, sweets, and ultra-processed products, while showing lower intake of fruits, vegetables, and fiber-rich foods [5,6,13,46,52]. These patterns suggest that screen exposure is not merely associated with isolated food choices, but with a broader shift toward a more energy-dense and nutrient-poor dietary profile.

A specific concern is the association between recreational screen exposure and ultra-processed foods [5]. These products are typically energy-dense, highly palatable, inexpensive, heavily marketed, and convenient for consumption during screen-based activities [53]. Their combination of high sugar, salt, fat, and refined carbohydrate content may promote passive overconsumption, while packaging, branding, and repeated digital cues may reinforce preferences from early childhood. For this reason, ultra-processed foods represent an important link between screen-related marketing environments and pediatric obesity risk.

Several factors may contribute to this association, including distracted eating, exposure to food advertising, convenience-driven food choices, irregular meal timing, reduced parental supervision during screen-based activities, and lower participation in structured family meals [18,20,23,41]. In addition, high recreational screen use often clusters with other lifestyle behaviors, including lower physical activity, sedentary behavior, irregular sleep, and less favorable household routines [16,17,54,55]. Similar associations between dietary habits, nutritional knowledge, and lifestyle behaviors have also been described in adolescent populations [56].

The relationship between screen exposure and dietary quality appears to be bidirectional and socially patterned. Household environment, parental education, socioeconomic status, and family eating routines may influence both media exposure and food choices [22,23,33,57]. Importantly, unhealthy dietary habits established during childhood frequently persist into adolescence and adulthood, potentially increasing long-term cardiometabolic risk [11,12,17,54]. This concept is consistent with epidemiological evidence highlighting the long-term impact of early-life lifestyle behaviors on obesity risk and metabolic health [57,58].

### 4.4. Irregular Eating Patterns and Meal Disruption

Several studies have described associations between prolonged screen time and increased snacking frequency, irregular meal schedules, and meal skipping, particularly breakfast skipping [5,6,59].

Screen-based activities often promote grazing behaviors characterized by repetitive consumption of small portions of highly palatable foods throughout the day. In many cases, snacking occurs simultaneously with digital media use, reducing awareness of food intake and encouraging passive overconsumption [5,41,46]. This pattern may be particularly relevant when screen use is accompanied by highly palatable, energy-dense foods, which are commonly marketed and consumed during screen-based activities [41,44,50].

Irregular meal timing and breakfast skipping may have important metabolic implications. Breakfast omission has been associated with poorer dietary quality, increased hunger later in the day, and greater likelihood of consuming energy-dense foods [2,3,5]. Furthermore, late-night screen use may delay sleep timing and prolong waking hours, increasing opportunities for nighttime eating and circadian misalignment [1,2,6,59].

Breakfast skipping deserves particular attention because it is a recurrent marker of irregular eating patterns in school-aged children and adolescents [60]. Children who skip breakfast may have lower overall diet quality, reduced intake of fiber and micronutrient-rich foods, greater hunger later in the day, and higher consumption of energy-dense snacks or sugar-sweetened beverages [60,61]. Late-night screen use may contribute indirectly by delaying bedtime, shortening sleep duration, increasing morning fatigue, and reducing time or appetite for breakfast [53]. However, breakfast skipping should not be interpreted as a direct consequence of screen exposure in all children, because it may also reflect family routines, school schedules, socioeconomic factors, body image concerns, dieting behaviors, or emotional distress.

Future studies should distinguish occasional from habitual breakfast skipping, weekday from weekend patterns, and nutritionally adequate breakfasts from energy-dense breakfast substitutes. This distinction is important because the health implications of breakfast patterns depend not only on whether breakfast is consumed, but also on food quality, timing, family context, sleep duration, and total daily dietary intake.

These altered eating behaviors may act together with sedentary behavior, sleep disturbances, and neurocognitive mechanisms involved in appetite regulation, thereby increasing susceptibility to weight gain and metabolic dysfunction [16,18,43,45,46]. However, as most available evidence is observational, it remains difficult to determine whether screen exposure directly disrupts meal timing or whether both behaviors reflect broader household routines, lifestyle patterns, and family-level determinants.

## 5. Biological and Behavioral Mechanisms

### 5.1. Sedentary Behavior

Sedentary behavior (SB) refers to any waking activity performed with minimal energy expenditure (<1.5 metabolic equivalents), such as sitting or lying down [16,17]. In addition to reducing daily energy expenditure, high levels of sedentary behavior are associated from early life with poorer cardiovascular health, lower fitness, and reduced quality of life [17,18].

Increased sedentary time and low levels of physical activity are contributing factors to the development of non-communicable diseases, as well as to the rising global prevalence of obesity and metabolic syndrome (MetS). Indeed, the worldwide prevalence of MetS among children and adolescents in 2020 was 2.8% and 4.8%, respectively, and it is expected to increase in the coming years as obesity rates continue to rise [54,62].

Recent surveillance data indicate that 81% of children and adolescents aged 11–17 years do not meet recommended physical activity guidelines [16,17,21]. Physical inactivity is estimated to contribute to nearly 500 million new preventable non-communicable disease cases. Globally, its annual economic burden is approximately US$27 billion and is projected to exceed US$300 billion by 2030 [17].

Sedentary behavior has multifactorial determinants, including socioeconomic status, sex, and age [22]. Blyth and colleagues reported healthier habits among children and adolescents from higher socioeconomic backgrounds compared with those from lower socioeconomic groups [22,23]. These findings also suggest that behaviors established early in life tend to remain stable over time, highlighting the importance of promoting healthy routines from early childhood [22,24].

Over recent decades, digital technologies have become embedded in children’s and adolescents’ everyday lives. Personal smartphones and social media have contributed to rising screen time among young populations [25]. At the same time, some studies have explored smartphone-based interventions to promote physical activity, using reward systems and engagement strategies [26,27].

The “Pokémon Go” app gained immediate popularity among children, adolescents and adults, gathering the attention of both media and the scientific community even though it was not intentionally developed for health purposes [63]. A systematic review and meta-analysis found that playing “Pokémon Go” significantly increased the total amount of daily steps; however, these improvements were deemed modest from a clinical perspective [64], and follow-up investigations failed to show that users maintained this behavior in the long term [63,64].

As far as sedentary behavior is concerned, implementing physically active screen time in children’s and adolescents’ daily routines does not appear to significantly reduce overall sedentary time or mitigate its negative effects on health status [55,65].

Screen time is typically associated with increased sedentary behavior, as most screen-based activities are performed while sitting, reclining, or lying down [54,66]. In 2018, Shakir and colleagues investigated how different types of sedentary behavior influence health outcomes [67]. Their findings showed that watching television was significantly associated with greater adiposity indices in both boys and girls, with children spending an estimated time of 2.5–3 h per day in television viewing. Computer screen time was also positively associated with greater fat mass percentages in both sexes. Furthermore, the study suggested that television screen time was the strongest correlate of adiposity in girls, whereas computer screen time appeared to be the most significant factor in boys, likely due to video-gaming habits being more prevalent among boys than girls.

Recent literature further supports the hypothesis that, among the various forms of sedentary behavior, screen time showed stronger links to unhealthy body compositions and weight [46]. Systematic reviews and meta-analyses have also highlighted a positive association between screen time and the presence of MetS in children and adolescents. Regardless of the type of screen-based activity, time spent in front of a screen is associated with increased consumption of calorie-dense food and beverages among youth [46,52,55].

Beyond physical health, evidence suggests that screen time and sedentary behavior in adolescents are associated with poorer mental health, reduced sleep quality and duration, lower energy levels, irritability, somnolence, and sadness [68]. International sedentary-behavior guidelines report that high recreational screen exposure and prolonged sedentary time are associated with less favorable sleep and mental health indicators in children and adolescents [69]; therefore, the cited guideline source has been verified and should be aligned with the final reference list.

Despite the extensive evidence, specific dose–response guidelines regarding sedentary behavior and the risk of developing MetS are still lacking [46]. Several countries have issued recommendations to limit sedentary time to approximately 2 h per day, whereas the WHO recommends more generally limiting time spent in sedentary activities [70,71].

Future longitudinal studies should investigate the role of physical inactivity and its relationship with the development of MetS to provide clearer guidance on appropriate sedentary behavior limits in children and adolescents [46,47,72].

### 5.2. Neurocognitive Dysregulation

Screen-based activities are associated with increased energy intake and reduced energy expenditure, as they are predominantly part of sedentary behavior [41,46].

Food companies capitalize on this trend by strategically placing advertisements for energy-dense foods and beverages across popular social media and streaming platforms which are widely used by older children and adolescents [41,50]. Similarly, television, particularly prevalent among children aged 4–7, frequently exposes young users to repeated food commercials [50].

Compared to younger children, adolescents progressively gain greater autonomy over their meal choices. Nonetheless, adolescence is also a particularly vulnerable developmental stage, during which individuals are highly susceptible to peer pressure and social influences [50]. From a neurobiological perspective, adolescence is characterized by reduced inhibitory control and higher reward drive compared to adulthood [51]. Given that inhibitory processes are still maturing during this period [43], adolescents may be especially vulnerable to food advertisements that exploit these neurophysiological mechanisms for marketing purposes [41,44]. Scientific evidence has shown that visually appealing food commercials can activate dopaminergic mesolimbic pathways in the brain, which are closely linked to reward processing and may influence food intake and preferences [44].

Inhibitory control mechanisms are essential for resisting impulsive choices (e.g., binge-eating episodes) [45], and increased screen exposure has been shown to negatively affect these neurological pathways. Chen and colleagues reported that preadolescents with high daily screen time exhibited altered connectivity within the fronto-striatal network, which was associated with reduced inhibitory efficiency. Furthermore, their 2-year longitudinal study demonstrated that greater screen exposure was linked to decreased connectivity between the frontoparietal network and the dorsal striatum. These neural alterations are often observed in addictive and reward-seeking behaviors [43]. Given their design, digital platforms offer immediate reward and feedback while delivering tailored content based on users’ preferences, which may further reinforce addictive patterns [43,44,66].

These neurocognitive findings should be interpreted cautiously. Neuroimaging and behavioral studies suggest plausible pathways linking reward sensitivity, food cue reactivity, inhibitory control, and impulsive eating, but they do not demonstrate that screen exposure alone causes neurocognitive dysregulation [73]. Screen use may also be a marker of broader developmental, psychological, family, and environmental factors that influence both media behavior and eating regulation.

The same neural pathways are also implicated in appetite regulation. Subcortical systems are heavily involved in shaping daily food preferences [43,45]. Evidence suggests that individuals with poorer inhibitory control are more likely to eat in the absence of hunger [42]. For example, Amens and colleagues reported that adolescents with impaired inhibitory control showed a greater tendency for binge-eating episodes.

In addition to these neurological mechanisms, trends promoted by streaming and social media platforms may further encourage snacking of calorie-dense food, especially at night [51]. This behavior may disrupt regular meal routines and is associated with greater odds of overweight and obesity. Moreover, late-night snacking may delay bedtime and can negatively affect sleeping patterns [51,59].

Previous studies have also found that children and adolescents with overweight and obesity exhibit reduced inhibitory response to food cues, suggesting a distinct neurobiological pattern associated with obesity [45,48]. In light of these findings, current trends in excessive screen exposure, sedentary lifestyles, and availability of energy-dense foods may be associated with an increased risk of obesity in children and adolescents [41,74].

### 5.3. Sleep and Circadian Rhythms

Sleep is a fundamental biological process for physical and mental recovery. It supports brain information processing, homeostasis, and neuroendocrine regulation; when sleep duration is shortened or timing is delayed, these functions may be impaired, contributing to circadian misalignment and metabolic dysregulation [9,10].

Sleep disturbances and circadian rhythm disruption are key mechanisms linking excessive screen time with unhealthy eating behaviors and adverse health outcomes in children. Insufficient sleep and circadian misalignment have been associated with obesity, insulin resistance, and cardiometabolic risk in pediatric populations [1,3,5,6].

Circadian rhythms regulate hormone secretion, energy metabolism, and feeding behavior through the interaction between central and peripheral clocks. Irregular sleep schedules and excessive evening screen time can disrupt these rhythms, altering appetite control, glucose metabolism, insulin sensitivity, and body fat regulation [5,6].

Screen-based behaviors, particularly in the evening, may delay sleep timing and reduce sleep duration. These changes can promote irregular eating patterns, increased snacking, and preference for energy-dense foods, while also interacting with neuroendocrine and reward-related pathways that reinforce unhealthy eating and susceptibility to weight gain [2,5,6].

However, the available evidence remains heterogeneous in terms of study design, population characteristics, and measurement of both screen exposure and sleep outcomes, which should be considered when interpreting these findings.

#### 5.3.1. Evening Screen Time and Sleep Duration

Evening screen exposure has been consistently associated with reduced sleep duration and impaired sleep quality in pediatric populations. The use of digital devices before bedtime is associated with delayed sleep onset, shorter total sleep time and increased sleep fragmentation. These effects are mediated by both physiological and behavioral mechanisms [75].

From a physiological perspective, exposure to artificial light emitted by screens interferes with melatonin secretion, disrupting the circadian regulation of the sleep–wake cycle and contributing to delayed sleep onset. From a behavioral standpoint, engaging and stimulating screen-based activities may increase cognitive and emotional arousal, further delaying bedtime and displacing sleep time [76,77].

Recent experimental evidence supports a potential causal role of pre-sleep screen exposure in sleep disruption. A randomized clinical trial showed that removing screen use during the hour before bedtime improved sleep duration, sleep efficiency, and nighttime awakenings in children [4], suggesting that even short-term changes in evening screen habits may benefit sleep health.

#### 5.3.2. Hormonal Alterations: Leptin and Ghrelin

The relationship between evening screen exposure, sleep disruption, appetite-related hormones, and energy balance should be interpreted as a plausible mechanism rather than as a simple causal chain. The magnitude of these effects is likely to vary according to age, pubertal stage, sleep regularity, device type, content, and household routines.

Evening screen exposure, by delaying bedtime and reducing total sleep duration, can lead to dysregulation of appetite-related hormones, particularly leptin and ghrelin, which are essential for energy balance [78]. Leptin, an anorexigenic hormone, signals satiety and reduces food intake, whereas ghrelin, an orexigenic hormone, stimulates hunger and promotes food consumption. Both hormones are sensitive to sleep duration and circadian timing, and their secretion follows a circadian rhythm [76,79,80].

Insufficient sleep resulting from prolonged evening screen use has been associated with decreased leptin levels and increased ghrelin levels, amplifying hunger and the drive to eat, especially energy-dense and palatable foods. These neuroendocrine changes provide a mechanistic explanation for the observed association between late-night screen use, increased snacking, irregular meal patterns and higher caloric intake in children [76,80]. Experimental evidence suggests that even modest reductions in sleep, such as those caused by evening screen exposure, can significantly disturb leptin and ghrelin balance, contributing to a positive energy balance and increased risk of weight gain [81].

Moreover, by prolonging the period during which children are awake, evening screen exposure increases opportunities for late-night eating, further reinforcing unhealthy dietary behaviors. The combination of altered hormone signaling and extended eating windows may synergistically contribute to the development of overweight and obesity. Circadian misalignment due to delayed sleep also impairs metabolic regulation, including glucose tolerance and insulin sensitivity, thereby increasing long-term cardiometabolic risk [13,77].

## 6. Associated Health Risks

### 6.1. Childhood Obesity

Childhood obesity is one of the most consistently documented adverse outcomes associated with screen exposure. The relationship is multifactorial, involving increased energy intake during screen viewing, exposure to marketing of energy-dense and nutrient-poor foods, and reduced sleep duration, all of which may contribute to positive energy balance [10,82]. Supporting this framework, Presta et al. [1] in a systematic review including 75,540 children across 40 studies, reported a significant impact of screen exposure on adiposity parameters. Similarly, the umbrella review by Stiglic et al. [83] reported moderately strong evidence linking screen time to increased adiposity, with moderate evidence for higher energy intake and poorer diet quality. Consistent findings have also been reported in younger children, including associations between excessive screen time and overweight/obesity, higher body mass index, abdominal adiposity, and skinfold thickness [84,85].

More recently, a large meta-analysis by Jiang et al. [86], encompassing 419 studies, found that more than 2 h of daily screen time was associated with increased childhood obesity risk. This finding is consistent with subsequent evidence, including a Chinese systematic review and meta-analysis published in 2026 [87] and data from Sub-Saharan African preschool populations [88].

Longitudinal studies provide further support for a temporal relationship between screen exposure and obesity risk. Early long-term cohort studies have shown that greater television viewing during childhood predicts overweight and obesity in adulthood.

In a more recent longitudinal study by Goodman et al., including 16,376 children in the UK, video game use at age 5 years was associated with a higher body mass index SD score at age 14 years [89]. Likewise, Nagata et al. showed that each additional hour of daily screen time among children aged 9–10 years was associated with a higher BMI percentile after one year; texting, video chat, and video games showed significant associations when specific behaviors were examined [90].

Consistent with these results, in a longitudinal study by Miguel-Berges et al. on 718 children from six European countries, sedentary lifestyle including television viewing and computer game use were significantly associated with increased risk of overweight and obesity [91].

A dose–response relationship between screen time and adiposity is consistent with a possible pathway. In a review of 73 studies, Carson et al. reported that higher screen exposure was consistently associated with less favorable body composition [92]. Similar dose–response patterns have been observed for television viewing [93,94]. Quantitative estimates from Fang et al. [95] indicate a 67% higher obesity risk among children engaging in ≥2 h of screen time per day compared with those below this threshold, with consistent associations across screen modalities.

A cross-sectional study on 5797 adolescents also supports this gradient, showing that medium (4–8 h per day) or high (more than 8 h per day) levels of screen time, compared with low levels (less than 4 h per day), were associated with higher BMI percentile and overweight or obesity. A dose–response association was observed, and it was not attenuated by higher physical activity [96]. Similarly, Nightingale et al.’s survey of 4495 children aged 9–10 years found that those with more than 3 h of daily screen time had higher ponderal index values (calculated by weight divided by height cubed), compared with those with less than 1 h per day [97].

Additional evidence suggests that contextual factors may further exacerbate risk. For instance, in a cross-sectional analysis of 630 Canadian children aged 8–10 years, screen exposure exceeding 2 h per day has been associated with higher energy intake and lower consumption of fiber, fruits, and vegetables, particularly among overweight children [6]. Environmental and behavioral factors, such as the presence of a television in the bedroom or unrestricted nighttime access to electronic devices, have also been linked to increased obesity risk [98,99,100]. Moreover, parenting practices appear to play a role: the use of screen time as a reward has been associated with higher BMI z-scores in children, whereas monitoring and limiting screen use is linked to more favorable weight outcomes [101].

Despite the overall consistency of findings, some studies have reported null associations [102,103,104]. For example, Figueira et al. [42] evaluated 4531 children at 4 and 7 years but no statistically significant differences were found regarding changes in screen time and the child’s BMI. Veldman et al. [2] found no evidence of association of screen time and BMI in the early years (0–5 years), while Myszkowska-Ryciak et al. [14] found no significant relationship between screen time and body weight status in Polish adolescents. These discrepancies may reflect differences in age groups, measurement methods, or confounding factors, but do not substantially weaken the broader body of evidence supporting a positive association between screen time and childhood obesity.

The evidence should therefore be interpreted as strongest for an association between high recreational screen exposure and unfavorable adiposity indicators, while causality remains more difficult to establish. Potential bidirectionality is important: children with overweight, low physical activity, sleep problems, emotional distress, or fewer structured routines may also be more likely to engage in prolonged screen use. Differences in exposure measurement, residual confounding, and the distinction between television, gaming, social media, streaming, and educational screen use may partly explain inconsistent findings.

### 6.2. Cardiometabolic Risk

A growing body of evidence indicates that excessive screen time is associated not only with obesity but also with a broader spectrum of cardiometabolic risk factors in children and adolescents, including hypertension, dyslipidemia, insulin resistance, systemic inflammation, and MetS. Observational studies consistently support these associations [105,106,107]. A systematic review by Musa et al. reported a significant association between screen time of any type and MetS among adolescents, with approximately 70% of the included studies demonstrating a dose–response gradient [108].

For instance, Horner et al. [109], analyzing over 1000 participants from two mother–child cohorts, found that each additional hour of daily screen time was associated with higher cardiometabolic risk in both children and adolescents. Similarly, Kunin et al. [110] observed that children with more than 2 h of average daily screen exposure had a significantly higher likelihood of elevated cardiometabolic risk. Cross-sectional evidence aligns with these results: metabolically unhealthy adolescents have been shown to report longer screen time compared with their healthy counterparts, and environmental factors such as the presence of a television in the bedroom have been associated with increased cardiometabolic risk and elevated triglyceride levels [100,111].

Additional data suggest that different types of screen use may have distinct effects; for example, leisure-time computer and video game use has been linked to higher overall cardiometabolic risk scores, while television viewing appears to have sex-specific associations [112]. Moreover, screen time may interact with individual susceptibility, as it has been shown to moderate the relationship between genetic predisposition to obesity and cardiometabolic risk, particularly in youth with low cardiorespiratory fitness [113]. Nevertheless, some longitudinal findings remain inconsistent, with certain studies reporting no significant association between screen exposure and MetS over time, highlighting the complexity of these relationships [54].

#### 6.2.1. Insulin Resistance

Insulin Resistance, a state when cells fail to use insulin effectively, contributes to the pathophysiology of type 2 diabetes and is a risk factor for cardiovascular diseases. Evidence suggests that excessive screen time is associated with poorer insulin sensitivity in pediatric populations. Hardy et al. showed that adolescent boys engaging in ≥2 h of weekday screen time have twice the risk of abnormal levels of insulin and HOMA-IR compared with those with lower exposure [114]. Similarly, Henderson et al. showed that high screen time predicted worse insulin sensitivity in a two-year follow-up, although obesity partially mediated these results [115]. Furthermore, large cross-sectional data from Nightingale et al. showed that children aged 9–10 years reporting more than 3 h of daily screen time exhibited significantly higher levels of insulin resistance compared with those with ≤1 h per day, even after adjustment for adiposity, socioeconomic factors, and physical activity [97].

#### 6.2.2. Dyslipidemia

Screen time has also been linked to adverse lipid profiles, particularly reduced high-density lipoprotein (HDL) cholesterol levels. Martinez-Gomez et al. [107], in a cross-sectional study of 425 adolescents, concluded that screen time of more than three hours was associated with a significant decrease in HDL cholesterol. Consistent with this, Van Ekris et al. identified moderate-to-strong evidence for an inverse association between sedentary behavior, including screen time, and HDL levels, while Goldfield et al. reported similar findings among obese adolescents engaged in video gaming [106,116].

Longitudinal evidence further supports this relationship: Vanderloo et al. [117] observed an inverse association between screen time and HDL cholesterol in children, although no significant associations were found with other cardiometabolic components.

In younger children, findings are less consistent; while some studies report no association between screen time and overall cardiometabolic risk, small but significant relationships with non-HDL cholesterol have been identified [118]. Notably, Berentzen et al. demonstrated that the association between screen time and lipid ratios may be largely mediated by adiposity, suggesting that indirect pathways play a substantial role [119].

#### 6.2.3. Hypertension

An increasing number of studies have examined the relationship between screen exposure and blood pressure (BP) in pediatric populations. Evidence suggests that both the duration and type of screen use may be associated with BP levels. In an observational study of 546 children with obesity, Pardee et al. reported that daily television viewing, together with obesity severity, was an independent predictor of hypertension [120]. Similarly, Martinez-Gomez et al. found that television viewing and total screen time, but not computer use, were positively associated with BP independently of body composition [121]. Cross-sectional data also indicate that heavy internet use may be associated with higher odds of elevated BP among adolescents [122]. Longitudinal findings further support these associations: Gopinath et al., after adjusting for BMI and other confounding factors, found that in a pediatric population, each hour per day spent in total screen time was associated with an increase in diastolic BP and mean arterial BP, with particularly pronounced effects observed for television viewing in boys over a five-year period [123]. Consistent results have been reported in other cohorts, where screen-based behaviors were positively associated with diastolic BP across adolescence [124]. More recent studies continue to corroborate these findings, linking high screen exposure to elevated systolic BP in both general pediatric populations and in children with chronic conditions such as type 1 diabetes [125,126].

Overall, while some inconsistencies remain, the available evidence supports an association between excessive screen time and adverse cardiometabolic outcomes in children, with effects reported across multiple physiological domains.

Table 1 summarizes the main studies on screen exposure and cardiometabolic outcomes in children and adolescents. Overall, associations appear stronger when screen time co-occurs with adiposity, low physical activity, poor diet quality, or insufficient sleep; therefore, future studies should account for these lifestyle and contextual factors as potential confounders, mediators, or effect modifiers.

### 6.3. Mental Health

Mental health outcomes should be clearly distinguished from disordered eating constructs and formal eating disorder diagnoses. Anxiety and depressive symptoms, emotional eating, binge-eating symptoms, body dissatisfaction, and DSM-defined eating disorders are related but distinct conditions. The relationships between screen exposure and these outcomes should be interpreted cautiously, as they may be bidirectional and influenced by sleep, social comparison, cyberbullying, emotional regulation, physical activity, and family or peer context.

#### 6.3.1. Anxiety and Depression

Over recent years, screen time has become increasingly prevalent, with an expanding variety of electronic media devices available worldwide. Screen time, in particular, television viewing, has been negatively associated with the development of physical and cognitive abilities, and positively associated with obesity, sleep problems, depression and anxiety. The physiological mechanisms that underlie the adverse health outcomes related to screen time and the relative contributions of different types of screen and media content to specific health outcomes are still unclear [127]. A Brazilian study published in 2024 found consistent evidence of an association between depressive symptoms and recreational screen time among adolescents. One possible pathway is the displacement of face-to-face interpersonal relationships by screen-based activities, which may increase social isolation and depressive symptoms. Conversely, physical activity and other structured leisure activities may reduce time spent in a sedentary screen-based setting [71]. However, these associations should be interpreted cautiously because most studies are observational and may be affected by bidirectionality and residual confounding.

Nagata et al. [96] reported that recreational screen time is associated with depressive symptoms in adolescents. However, much of the available evidence has important limitations. Many studies consider adolescents as a single group, without adequately examining whether associations differ by age, sex, ethnicity, socioeconomic background, or developmental stage. This limits the possibility of developing targeted prevention strategies for specific adolescent subgroups. Another important limitation is that many studies do not distinguish sufficiently between different screen-based activities. The association with depressive symptoms may vary according to whether screen use involves video chatting, texting, passive video viewing, gaming, or social media interaction. Therefore, total screen time alone may be an oversimplified measure.

Similarly, Santos et al. [128] emphasized that the concept of “screen time” does not adequately capture the type of content, the purpose of use, or the way adolescents interact with digital media. Future studies should consider not only duration, but also context, intensity, content, sleep, physical activity, socioeconomic factors, and individual vulnerability. Overall, the available findings suggest that the mental health implications of screen use depend on multiple interacting factors rather than screen duration alone.

#### 6.3.2. Emotional Eating

Emotional eating refers to eating in response to negative emotions rather than physiological hunger and should be distinguished from clinical eating disorders [129]. Screen exposure may be associated with emotional eating through several pathways, including sleep disruption, stress, mood symptoms, exposure to food cues, and the use of digital media for emotion regulation.

Eating disorders (EDs) encompass a group of psychiatric conditions characterized by persistent disturbances in eating behaviors that can result in significant physical, psychological, and social morbidity. According to the DSM-5, eating disorders include anorexia nervosa, bulimia nervosa, binge-eating disorder, other specified feeding and eating disorders, and avoidant/restrictive food intake disorder. Although these conditions may share some behavioral features, such as binge eating or food restriction, they should be considered distinct from emotional eating unless diagnostic criteria are met.

Subclinical eating-related behaviors, including uncontrolled eating, cognitive restraint, and emotional eating, may also be relevant in the context of screen exposure. Keeler et al. reported a positive association between screen time and these eating-related behaviors [130]. Smartphone addiction has also been linked to emotional eating, potentially through poor sleep quality and emotional dysregulation, which may increase impulsive eating in vulnerable individuals [131].

Regarding the sleep–wake cycle, Zhou et al. [132] examined the relationship between sleep quality and emotional eating among college students using a moderated mediation model in which depression acted as a mediator and physical activity as a moderator. Because this study involved college students rather than children or adolescents, it is cited only as indirect contextual evidence and should not be interpreted as equivalent to pediatric evidence. Its findings suggest that poor sleep quality is associated with higher emotional eating, partly through depressive symptoms, whereas physical activity may attenuate the association between depression and emotional eating. Poor sleep quality may also interact with smartphone addiction and emotional dysregulation, creating a potential cycle linking sleep disturbance, problematic smartphone use, and emotional eating.

### 6.4. Eating Disorders

#### 6.4.1. Early Binge Eating

Binge-eating disorder (BED) is characterized in the DSM-5-TR by recurrent binge-eating episodes, defined as consumption of an objectively large amount of food within a discrete period of time, accompanied by marked distress and occurring at least once per week for three months, in the absence of regular compensatory behaviors such as laxative abuse or self-induced vomiting.

A recent longitudinal study examined how different patterns of digital engagement are associated with binge-eating symptoms in adolescents over time [133]. The study identified two profiles: high screen use and low screen use, assessed at Time 3 (ages 10–13 years) and Time 5 (ages 12–16 years) [133]. Adolescents with more intensive and unbalanced digital-engagement profiles were more likely to develop or maintain binge-eating behaviors; in particular, high screen use at Time 5 was associated with greater risk. Importantly, the study highlights that not all forms of digital engagement are equally harmful: the type of activity and the way adolescents interact with digital content play a crucial role. This supports the view that measuring screen time alone is insufficient to understand mental-health outcomes. The findings also suggest that digital engagement may influence eating behaviors through psychological pathways, such as emotional-regulation difficulties or exposure to harmful online content. Overall, the study emphasizes the need for more nuanced research and targeted interventions that consider specific patterns of digital engagement rather than focusing only on duration.

In addition to the direct association between binge-eating disorder and screen time, a 2024 article in the International Journal of Eating Disorders examined the role of depression in this relationship, suggesting that excessive screen use may contribute to depressive symptoms, which in turn increase the likelihood of binge-eating behaviors [134].

#### 6.4.2. Body Image Issues

Body image concerns vary according to age and developmental stage [135]. In adolescents, screen exposure may be relevant through mechanisms such as appearance-based social comparison, internalization of idealized body standards, exposure to edited or filtered images, influencer content, peer feedback, and weight-related stigma [136,137]. These factors may contribute to body dissatisfaction or disordered eating symptoms, but they should not be equated with formal eating disorder diagnoses unless clinical diagnostic criteria are assessed.

The current literature on screen time and body image predominantly focuses on adolescents and emerging adults, whereas studies involving children mainly investigate cognitive, behavioral, and physical health outcomes rather than body dissatisfaction or appearance-related concerns [138]. Evidence from emerging adults is therefore considered only as indirect contextual background when pediatric data are limited.

Studies conducted in young adults and emerging adults suggest that screen-based media exposure may be associated with upward social comparison, internalization of idealized body standards, and body dissatisfaction [139]. These findings are not presented as pediatric evidence, but only as indirect background for interpreting possible developmental pathways that require confirmation in children and adolescents.

Another study conducted in 2022 examined associations between total screen time, viewing modes, body dissatisfaction, disordered eating patterns, and intentions to undergo cosmetic surgery [140]. Because the study population was not restricted to children and adolescents, its findings should be interpreted as indirect contextual evidence rather than direct evidence for the 0–18-year target population.

## 7. Implications and Prevention Strategies

Excessive screen exposure and its association with unhealthy eating behaviors highlight the need for integrated prevention strategies targeting children, adolescents, families, schools, and broader societal environments. Given the multifactorial nature of screen-related dietary and metabolic risks, isolated interventions are unlikely to be sufficient, whereas multidimensional and multidisciplinary approaches may provide more effective and sustainable outcomes [141,142]. Prevention strategies can be organized across five interconnected levels: individual counseling, family routines, school environments, community support, and regulatory policy.

### 7.1. Family-Based Interventions

Family environment plays a central role in shaping children’s screen habits, eating behaviors, and daily routines. Parents and caregivers influence screen exposure through modeling, household rules, supervision of digital content, and the organization of meals and sleep routines; therefore, family-based strategies should address both screen duration and the quality, timing, and context of media use. Practical targets include screen-free meals, removal or restriction of bedroom devices, consistent bedtime routines, parental modeling of balanced media use, avoidance of screens as rewards or emotion-regulation tools, and structured availability of healthy foods during leisure time [23,24,28]. These approaches should be adapted to family resources and should avoid stigmatizing children with overweight or obesity.

### 7.2. School-Based Interventions

Schools represent a key setting for promoting healthy dietary behaviors and reducing screen-related obesogenic behaviors among children and adolescents. School-based strategies should combine nutrition education, digital and media literacy, healthier school food environments, cafeteria nudging, restrictions on sugar-sweetened beverages, and regular opportunities for physical activity [141]. Students should learn to identify sponsored content, influencer marketing, advergames, algorithmic recommendations, and persuasive techniques used to promote ultra-processed foods. Programs should remain feasible, age-appropriate, and connected with family and community actions.

### 7.3. Public Health Policies

Policy-level actions should address the commercial determinants of child nutrition, including targeted advertising, influencer disclosures, platform accountability, age-appropriate advertising literacy, and restrictions on marketing energy-dense nutrient-poor foods to minors. They should also promote healthier school and community food environments and explicitly consider socioeconomic inequalities, because children in vulnerable households may experience higher exposure to unhealthy food environments and fewer opportunities for structured recreation or healthy meals [22,41,44].

### 7.4. Integrated and Multidisciplinary Approaches

The complex interaction between screen exposure, dietary behaviors, sedentary lifestyles, sleep disturbances, mental health, and metabolic health requires integrated and multidisciplinary approaches involving healthcare professionals, educators, psychologists, families, and policymakers [16,43,45,46]. Prevention strategies should move beyond isolated recommendations on screen duration and address the broader behavioral and environmental context in which screen-related eating behaviors occur.

In clinical and community settings, healthcare professionals may play an important role in identifying children at higher risk of unhealthy screen-related eating behaviors, including those with excessive recreational screen use, frequent snacking, irregular meal timing, sleep disturbances, emotional eating, or overweight and obesity. Nutritional counseling should therefore be combined with behavioral support, sleep hygiene strategies, promotion of physical activity, and guidance on family media routines [1,2,6,59].

Emerging evidence suggests that childhood obesity prevention should move beyond weight-centered models toward more comprehensive frameworks addressing behavioral, psychological, environmental, and social determinants of health [143,144]. This perspective is particularly relevant in the context of screen exposure, where eating behaviors may be shaped by digital marketing, family routines, peer influence, sedentary behavior, and emotional regulation mechanisms [41,42,51].

Overall, effective prevention strategies should combine nutritional education, behavioral interventions, family engagement, healthier food environments, digital and media literacy, sleep promotion, physical activity, and public policy measures. Such integrated approaches may help address the multifaceted determinants of unhealthy eating behaviors associated with excessive screen exposure and support healthier developmental trajectories in pediatric populations [141,144].

## 8. Limitations and Future Research Directions

This review also has limitations. Because it was designed as a narrative review, it did not include a formal risk-of-bias assessment, pooled effect estimates, certainty grading, or a PRISMA-style flow diagram. Study inclusion may therefore be affected by selection bias, and the conclusions should be interpreted as an integrative synthesis rather than a quantitative estimate of effect size. The heterogeneity of screen exposure definitions, dietary outcomes, developmental stages, and study designs also limits direct comparison across studies.

The available evidence on the relationship between screen time, unhealthy eating behaviors, and health risks in children presents several limitations. First, a large proportion of studies is cross-sectional, limiting the possibility of establishing causal relationships. Although associations between excessive screen exposure, poorer dietary quality, increased snacking, obesity, cardiometabolic risk, sleep disturbances, and mental health outcomes are frequently reported, the directionality of these relationships remains difficult to determine.

A further limitation concerns the heterogeneity of screen time assessment. Studies often use different definitions, thresholds, questionnaires, and recall periods, making comparisons challenging. In addition, screen exposure, dietary intake, and eating behaviors are frequently assessed through self- or parent-reported measures, which may be affected by recall bias and social desirability bias.

Many studies also rely on total screen time without adequately distinguishing between different types, contexts, and purposes of use, such as television viewing, gaming, social media, streaming, messaging, or educational activities. This is particularly relevant because different screen-based activities may have distinct effects on food choices, snacking, sleep, physical activity, and mental health.

Another important challenge is the rapid evolution of digital platforms and media habits. The increasing use of smartphones, social media, influencer-generated content, streaming platforms, and algorithm-driven advertising may reduce comparability between older and more recent studies and calls for updated research designs reflecting contemporary digital environments.

It remains difficult to isolate the independent role of screen exposure from broader lifestyle, family, and socioeconomic factors. High screen time often co-occurs with sedentary behavior, low physical activity, irregular sleep, unstructured meals, parental media habits, unhealthy food availability, and socioeconomic disadvantage. Future studies should better account for confounders and mediators such as sleep quality, circadian disruption, mental health, family routines, and food environments.

Research should prioritize longitudinal and interventional designs to clarify causal pathways and evaluate family-based, school-based, and policy-level strategies. Key targets include screen-free meals, reduced evening exposure, digital and media literacy, and regulation of digital food marketing, particularly social media, influencer content, and algorithm-driven promotion.

Standardized, objective, and multidimensional tools are needed to assess screen exposure beyond duration, including content, context, timing, and purpose of use. Future studies should integrate dietary, metabolic, sleep-related, neurocognitive, neuroendocrine, inflammatory, microbiota-related, and mental health outcomes within unified study designs.

Greater attention should be given to vulnerable populations, socioeconomic inequalities, and culturally tailored prevention strategies to support more effective, precise, and equitable pediatric obesity prevention.

## 9. Summary

The evidence summarized in this review indicates that excessive and poorly contextualized screen exposure is associated with a cluster of unhealthy eating behaviors, including distracted eating, frequent snacking, irregular meals, breakfast skipping, and greater consumption of energy-dense and ultra-processed foods. The strength of evidence varies by outcome and study design, and many findings should be interpreted as associations rather than definitive causal relationships.

**Figure 1:**
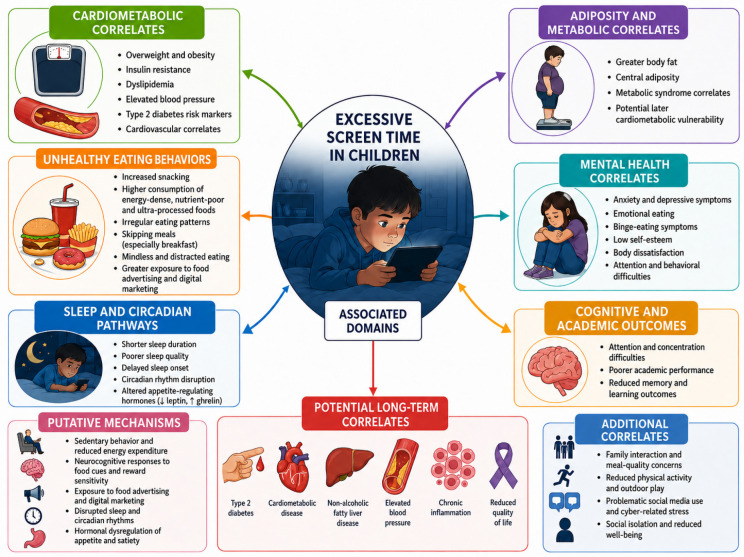
Summary of the main screen-related correlates and potential pathways associated with excessive screen time in children and adolescents. Prolonged screen exposure is associated with unhealthy eating behaviors, including increased snacking, mindless eating, irregular meal patterns, and higher consumption of ultra-processed foods. Excessive screen time may also be associated with sleep and circadian rhythm disturbances, sedentary behavior, putative neurocognitive mechanisms, and altered appetite-related hormonal regulation. These potential pathways may contribute to obesity, metabolic syndrome, insulin resistance, dyslipidemia, hypertension, and future cardiometabolic risk. In addition, excessive screen use has been linked to adverse mental health outcomes, including anxiety, depression, emotional eating, binge-eating behaviors, low self-esteem, and body image dissatisfaction, as well as poorer attention, cognitive, and academic outcomes. The figure was generated with the support of ChatGPT, version GPT-5.5 Thinking (OpenAI), following detailed instructions provided by the authors. The authors subsequently reviewed, edited, and validated the final figure to ensure consistency with the evidence discussed in the manuscript.

Figure 1 synthesizes the main pathways discussed in the review. It presents screen exposure as a multidimensional construct linked to dietary behaviors, sedentary time, sleep and circadian disruption, neurocognitive and appetite-regulation mechanisms, obesity, cardiometabolic risk, and mental health outcomes. The figure should be read as a conceptual summary of plausible and evidence-supported associations, not as proof of a single linear or deterministic causal pathway.

## 10. Conclusions

Screen exposure in childhood and adolescence may represent a modifiable behavioral and environmental correlate of dietary quality, metabolic health, sleep, and psychological wellbeing. Prevention should not focus only on reducing screen duration, but also on improving the timing, content, context, and purpose of digital media use.

Clinicians, families, schools, and policymakers should prioritize screen-free meals, reduced evening exposure, parental mediation, nutrition and media literacy, healthier food environments, physical activity, sleep hygiene, and regulation of digital food marketing directed at minors. Future research should use longitudinal and interventional designs, objective and multidimensional screen measures, developmental-stage-specific analyses, and careful control of confounding and mediation to clarify causal pathways and identify effective, equitable prevention strategies.

## Figures and Tables

**Table 1:** Summary of evidence linking screen time with cardiometabolic outcomes in children and adolescents.

First Author	Study Type/Population	Cardiometabolic Outcome	Main Findings	Interpretation
Presta et al. [1]	Systematic review; 75,540 children across 40 studies	Adiposity/obesity	Digital device exposure was associated with unfavorable adiposity parameters.	Supports an association between screen exposure and adiposity, although mechanisms are likely multifactorial.
Stiglic et al. [83]	Umbrella review	Adiposity, energy intake, diet quality	Reported moderately strong evidence linking screen time with increased adiposity and moderate evidence for higher energy intake and poorer diet quality.	Provides review-level evidence that screen time may be part of an obesogenic behavioral pattern.
Jiang et al. [86]	Large meta-analysis; 419 studies	Childhood obesity	More than 2 h/day of screen time was associated with increased childhood obesity risk.	Supports a dose-related association between screen exposure and obesity risk, although residual confounding cannot be excluded.
Goodman et al. [89]	Longitudinal cohort; 16,376 UK children	BMI	Video game use at age 5 years was associated with a higher BMI standard deviation score at age 14 years.	Suggests a possible long-term association between early screen-related behaviors and later adiposity.
Nagata et al. [90]	Longitudinal study; children aged 9–10 years	BMI percentile	Each additional hour/day of screen time was associated with a higher BMI percentile after one year; texting, video chat, and video games showed significant associations.	Indicates that specific screen activities may be differentially associated with adiposity.
Miguel-Berges et al. [91]	Longitudinal study; 718 children from six European countries	Overweight/obesity	Sedentary lifestyle, including television viewing and computer game use, was associated with increased risk of overweight and obesity.	Supports the relevance of sedentary screen behaviors in weight-gain trajectories, while potential confounding should be considered.
Carson et al. [92]	Systematic review of 73 studies	Body composition	Higher screen exposure was consistently associated with less favorable body composition.	Reinforces the association between recreational screen time and adiposity indicators.
Fang et al. [95]	Meta-analysis	Obesity risk	Children with ≥2 h/day of screen time had a 67% higher risk of obesity compared with those below this threshold.	Supports a threshold-based association, although residual confounding remains possible.
Nagata et al. [96]	Cross-sectional study; 5797 adolescents	Overweight and obesity	Medium and high screen time categories were associated with higher overweight or obesity risk compared with the low screen time category.	Suggests an association between higher screen time and overweight/obesity in adolescents; causality cannot be inferred from the cross-sectional design.
Nightingale et al. [97]	Cross-sectional study; 4495 children aged 9–10 years	Ponderal index, insulin resistance	Children with >3 h/day of screen time had higher ponderal index and higher insulin resistance than those with ≤1 h/day.	Suggests associations with adiposity and metabolic dysfunction, although residual confounding may remain.
Musa et al. [108]	Systematic review	Metabolic syndrome	Screen time of any type was associated with metabolic syndrome in adolescents; about 70% of included studies showed a dose–response gradient.	Provides review-level evidence for an association with clustered cardiometabolic risk, although the evidence is mostly observational.
Horner et al. [109]	Cohort analysis; >1000 participants from two mother–child cohorts	Global cardiometabolic risk	Each additional hour/day of screen time was associated with higher cardiometabolic risk in children and adolescents.	Supports a graded association between screen time and cardiometabolic risk.
Kunin et al. [110]	Pediatric observational study	Elevated cardiometabolic risk	Children with >2 h/day of screen exposure had a higher likelihood of elevated cardiometabolic risk.	Suggests that screen time thresholds may act as markers of increased cardiometabolic vulnerability.
Hardy et al. [114]	Cross-sectional study; adolescents	Insulin, HOMA-IR	Adolescent boys with ≥2 h/day of weekday screen time had about twice the risk of abnormal insulin and HOMA-IR levels.	Suggests an association between screen time and insulin resistance, particularly in boys; causality cannot be inferred.
Henderson et al. [115]	Two-year prospective longitudinal cohort study; 630 children	Insulin sensitivity	High screen time predicted worse insulin sensitivity, although obesity partly mediated the association.	Indicates that adiposity may act as an important mediator in the relationship between screen time and insulin sensitivity.
Martinez-Gomez et al. [107]	Cross-sectional study; 425 adolescents	HDL cholesterol	Screen time >3 h/day was associated with significantly lower HDL cholesterol.	Suggests an association with an unfavorable lipid profile, particularly reduced HDL cholesterol.
Van Ekris et al. [116]	Systematic review and meta-analysis	HDL cholesterol/sedentary behavior	Identified moderate-to-strong evidence for an inverse association between sedentary behavior, including screen time, and HDL cholesterol.	Supports a link between sedentary screen-related behaviors and unfavorable lipid markers.
Goldfield et al. [106]	Cross-sectional study; adolescents with obesity	HDL cholesterol/video gaming	Video gaming was associated with lower HDL cholesterol among adolescents with obesity.	Suggests that associations may be more evident in metabolically vulnerable groups; causality cannot be established.
Vanderloo et al. [117]	Longitudinal study; children aged 7–12 years	HDL cholesterol	Screen time was inversely associated with HDL cholesterol, although other cardiometabolic components were not consistently associated.	Provides partial support for lipid-related associations, with inconsistent findings across outcomes.
Berentzen et al. [119]	Pediatric cohort study	Lipid ratios	Associations between screen time and lipid ratios appeared largely mediated by adiposity.	Highlights the potential role of adiposity as a mediator rather than screen exposure alone.
Pardee et al. [120]	Observational study; 546 children with obesity	Hypertension	Daily television viewing, together with obesity severity, was an independent predictor of hypertension.	Suggests that television viewing may be associated with additional blood pressure risk among children with obesity.
Martinez-Gomez et al. [121]	Cross-sectional study	Blood pressure	Television viewing and total screen time, but not computer use, were positively associated with blood pressure independently of body composition.	Indicates that screen modality may influence blood pressure associations; causal inference is limited by the study design.
Gopinath et al. [123]	Longitudinal pediatric cohort study	Diastolic BP, mean arterial BP	Each additional hour/day of total screen time was associated with increases in diastolic and mean arterial blood pressure, especially for television viewing in boys.	Supports a longitudinal association between screen time and blood pressure, particularly for television viewing.

## Data Availability

No new data were created or analyzed in this study.

## Supplementary Materials

The following supporting information can be downloaded at: https://www.mdpi.com/article/10.3390/children13070887/s1, Table S1. Search strategy used across databases.

## References

[1] Presta V., Guarnieri A., Laurenti F., Mazzei S., Arcari M.L., Mirandola P., Vitale M., Chia M.Y.H., Condello G., Gobbi G. (2024). The Impact of Digital Devices on Children’s Health: A Systematic Literature Review. J. Funct. Morphol. Kinesiol..

[2] Veldman S.L.C., Altenburg T.M., Chinapaw M.J.M., Gubbels J.S. (2023). Correlates of Screen Time in the Early Years (0–5 Years): A Systematic Review. Prev. Med. Rep..

[3] Muppalla S.K., Vuppalapati S., Reddy Pulliahgaru A., Sreenivasulu H. (2023). Effects of Excessive Screen Time on Child Development: An Updated Review and Strategies for Management. Cureus.

[4] Vedechkina M., Borgonovi F. (2021). A Review of Evidence on the Role of Digital Technology in Shaping Attention and Cognitive Control in Children. Front. Psychol..

[5] Gomes G.M.D., Souza R.C.V., Santos T.N., Santos L.C. (2024). Screen Exposure in 4-Year-Old Children: Association with Development, Daily Habits, and Ultra-Processed Food Consumption. Int. J. Environ. Res. Public Health.

[6] Shang L., Wang J., O’Loughlin J., Tremblay A., Mathieu M.-È., Henderson M., Gray-Donald K. (2015). Screen Time Is Associated with Dietary Intake in Overweight Canadian Children. Prev. Med. Rep..

[7] Votsi I.C., Koutelidakis A.E. (2025). How Screen Time Affects Greek Schoolchildren’s Eating Habits and Functional Food Consumption?—A Cross-Sectional Study. Nutrients.

[8] Ares G., Antúnez L., Alcaire F., Natero V., Gugliucci V., Machín L., De León C., Otterbring T. (2025). Associations between Exposure to Digital Food Marketing and Food Consumption in Adolescence: A Cross-Sectional Study in an Emerging Country. BMC Public Health.

[9] Khan A., Feng J., Chachay V., Tsang J.H., Huang W.Y., Sit C.H.P., Minichiello V. (2025). Bytes and Bites: Social Media Use and Dietary Behaviours among Adolescents across 41 Countries. Pediatr. Res..

[10] Robinson T.N., Banda J.A., Hale L., Lu A.S., Fleming-Milici F., Calvert S.L., Wartella E. (2017). Screen Media Exposure and Obesity in Children and Adolescents. Pediatrics.

[11] Gispert-Llauradó M., Escribano J., Ferré N., Grote V., Koletzko B., Ambrosini G., Verduci E., Gruszfeld D., Xhonneux A., Luque V. (2025). Association between Early Dietary Patterns and Cardiometabolic Health at Age 8: A Confirmatory Analysis of the European Childhood Obesity Project. Nutr. J..

[12] Li W., Shen Y., Hu G. (2026). Lifestyle Factors and Cardiometabolic Risk. Chin. Med. J..

[13] Rocka A., Jasielska F., Madras D., Krawiec P., Pac-Kożuchowska E. (2022). The Impact of Digital Screen Time on Dietary Habits and Physical Activity in Children and Adolescents. Nutrients.

[14] Myszkowska-Ryciak J., Harton A., Lange E., Laskowski W., Wawrzyniak A., Hamulka J., Gajewska D. (2020). Reduced Screen Time Is Associated with Healthy Dietary Behaviors but Not Body Weight Status among Polish Adolescents. Report from the Wise Nutrition—Healthy Generation Project. Nutrients.

[15] Diniz D.G., Bento-Torres J., Da Costa V.O., Carvalho J.P.R., Tomás A.M., Galdino De Oliveira T.C., Soares F.C., De Macedo L.D.E.D., Jardim N.Y.V., Bento-Torres N.V.O. (2024). The Hidden Dangers of Sedentary Living: Insights into Molecular, Cellular, and Systemic Mechanisms. Int. J. Mol. Sci..

[16] Yousuf M.S., Harvey H.L., Parahoo S.K., Ziadeh B.S., Kilani M., Al-Kamil E. (2021). The Effects of Pediatric Primary Prevention Programs on Screen-Time and Reading Habits of Children in Jordan. Int. J. Child Care Educ. Policy.

[17] Bernard J.Y., Caron F., Salinier-Rolland C. (2022). Young Children and Screens: Guidelines for Intervention during the Perinatal Period from the French National College of Midwives. J. Midwifery Women’s Health.

[18] Barr R. (2022). Building Equitable Access and Inclusion for Children Growing up in the Digital Age. Policy Insights Behav. Brain Sci..

[19] Gupta P., Shah D., Bedi N., Galagali P., Dalwai S., Agrawal S., John J.J., Mahajan V., Meena P., Mittal H.G. (2022). Indian Academy of Pediatrics Guidelines on Screen Time and Digital Wellness in Infants, Children and Adolescents. Indian Pediatr..

[20] Wijaya S., Jhaveri S., Perry L.K., Barr R., Kucker S.C. (2025). Parent Personality, Child Temperament, and Digital Media: Pathways to Language Development in Early Childhood. Dev. Psychol..

[21] Lo C.K.M., Chan K.L., Chan E.W.W., Ho F.K., Ip P. (2024). Differential Associations between Quantity, Content, and Context of Screen Time, and Children’s Health-Related Quality of Life: A Two-Wave Study. Comput. Hum. Behav..

[22] Rodrigues D., Gama A., Machado-Rodrigues A.M., Nogueira H., Silva M.-R.G., Rosado-Marques V., Stamatakis E., Jago R., Padez C. (2021). Screen Media Use by Portuguese Children in 2009 and 2016: A Repeated Cross-Sectional Study. Ann. Hum. Biol..

[23] Rai J., Kuzik N., Carson V. (2022). Demographic, Parental and Home Environment Correlates of Traditional and Mobile Screen Time in Preschool-aged Children. Child.

[24] Wu Z., Yu J., Xu C. (2023). Does Screen Exposure Necessarily Relate to Behavior Problems? The Buffering Roles of Emotion Regulation and Caregiver Companionship. Early Child. Res. Q..

[25] Priftis N., Panagiotakos D. (2023). Screen Time and Its Health Consequences in Children and Adolescents. Children.

[26] Fan H., Yan J., Yang Z., Liang K., Chen S. (2022). Cross-Sectional Associations between Screen Time and the Selected Lifestyle Behaviors in Adolescents. Front. Public Health.

[27] Lammers S.M., Woods R.J., Brotherson S.E., Deal J.E., Platt C.A. (2022). Explaining Adherence to American Academy of Pediatrics Screen Time Recommendations with Caregiver Awareness and Parental Motivation Factors: Mixed Methods Study. JMIR Pediatr. Parent..

[28] ÇeliK E., Özer Y., Özcan S. (2021). Screen Time of Preschool Children in Relation to Their Parents Screen Usage Habits and Family Functions. Cukurova Med. J..

[29] McDaniel B.T., Rasmussen S., Reining L., Culp L., Deverell K. (2023). Pilot Study of a Screen-Free Week: Exploration of Changes in Parent and Child Screen Time, Parent Well-Being and Attitudes, and Parent-Child Relationship Quality. Hum. Behav. Emerg. Technol..

[30] Sá C., Vilar J., Magalhães P., Vasques C. (2022). Sleep Time, Tv/Video Games and Snack Consumption in Preschool Children—A Cross-Sectional Study. Retos.

[31] Dalene K.E., Kolle E., Steene-Johannessen J., Hansen B.H., Ekelund U., Grydeland M., Anderssen S.A., Tarp J. (2022). Device-Measured Sedentary Time in Norwegian Children and Adolescents in the Era of Ubiquitous Internet Access: Secular Changes between 2005, 2011 and 2018. Int. J. Epidemiol..

[32] Chinapong S., Amornsriwatanakul A. (2023). Prevalence of Sedentary Behavior and Factors Associated with Screen Time among Thai Youths Aged 14–17 Years: A Cross-Sectional Population-Based Survey. J. Health Sci. Med. Res..

[33] Kaur N., Gupta M., Malhi P., Grover S. (2022). Prevalence of Screen Time Among Children Aged 2 to 5 Years in Chandigarh, a North Indian Union Territory. J. Dev. Behav. Pediatr..

[34] Kerai S., Almas A., Guhn M., Forer B., Oberle E. (2022). Screen Time and Developmental Health: Results from an Early Childhood Study in Canada. BMC Public Health.

[35] Council on Communications and Media (2016). Media and Young Minds. Pediatrics.

[36] AAP Screen Time Guidelines. https://www.aap.org/en/patient-care/media-and-children/center-of-excellence-on-social-media-and-youth-mental-health/qa-portal/qa-portal-library/qa-portal-library-questions/screen-time-guidelines/.

[37] WHO World Health Organization (2019). Guidelines on Physical Activity, Sedentary Behaviour and Sleep for Children Under 5 Years of Age.

[38] Canadian Paediatric Society Canadian Paediatric Society (2017). Screen Time and Young Children: Promoting Health and Development in a Digital World. Paediatr. Child Health.

[39] McArthur B.A., Tough S., Madigan S. (2022). Screen Time and Developmental and Behavioral Outcomes for Preschool Children. Pediatr. Res..

[40] Canadian Paediatric Society, Digital Health Task Force, Ottawa, Ontario (2019). Digital Media: Promoting Healthy Screen Use in School-Aged Children and Adolescents. Paediatr. Child Health.

[41] Evans R., Christiansen P., Jones A., Finney J., Boyland E. (2025). The Impact of Food Marketing via Video Game Live Streaming on Snack Intake in Adolescents: A Randomised Controlled Trial. Public Health Nutr..

[42] Figueira M., Santos A.C., Gregório M.J., Araújo J. (2023). Changes in Screen Time from 4 to 7 Years of Age, Dietary Patterns and Obesity: Findings from the Generation XXI Birth Cohort. Nutr. Metab. Cardiovasc. Dis..

[43] Chen Y.-Y., Yim H., Lee T.-H. (2023). Negative Impact of Daily Screen Use on Inhibitory Control Network in Preadolescence: A Two-Year Follow-up Study. Dev. Cogn. Neurosci..

[44] Yeum D., Jimenez C.A., Emond J.A., Meyer M.L., Lansigan R.K., Carlson D.D., Ballarino G.A., Gilbert-Diamond D., Masterson T.D. (2023). Differential Neural Reward Reactivity in Response to Food Advertising Medium in Children. Front. Neurosci..

[45] Ames S.L., Kisbu-Sakarya Y., Reynolds K.D., Boyle S., Cappelli C., Cox M.G., Dust M., Grenard J.L., Mackinnon D.P., Stacy A.W. (2014). Inhibitory Control Effects in Adolescent Binge Eating and Consumption of Sugar-Sweetened Beverages and Snacks. Appetite.

[46] Georgoulis M., Grapsa I., Arnaoutis G., Karachaliou A., Panagiotakos D., Saltaouras G., Bathrellou E., Yannakoulia M., Dimitrakopoulos G., Kontogianni M.D. (2026). A Systematic Review and Meta-Analysis of Longitudinal Studies Exploring the Link Between Physical Inactivity and Indicators of Childhood Overweight/Obesity and Metabolically Unhealthy Obesity Risk in Western Countries. Curr. Obes. Rep..

[47] Ferguson C.J., Kaye L.K., Branley-Bell D., Markey P. (2025). There Is No Evidence That Time Spent on Social Media Is Correlated with Adolescent Mental Health Problems: Findings from a Meta-Analysis. Prof. Psychol. Res. Pract..

[48] Efraim M., Kirwan C.B., Muncy N.M., Tucker L.A., Kwon S., Bailey B.W. (2021). Acute After-School Screen Time in Children Decreases Impulse Control and Activation toward High-Calorie Food Stimuli in Brain Regions Related to Reward and Attention. Brain Imaging Behav..

[49] Cena H., Toselli A., Tedeschi S. (2003). Body Uneasiness in Overweight and Obese Italian Women Seeking Weightloss Treatment. Eat. Weight Disord..

[50] Rodríguez-Barniol M., Pujol-Busquets G., Bach-Faig A. (2024). Screen TimeUse and Ultra-Processed Food Consumption in Adolescents: A Focus Group Qualitative Study. J. Acad. Nutr. Diet..

[51] Domoff S.E., Sutherland E., Yokum S., Gearhardt A.N. (2021). The Association of Adolescents’ Television Viewing with Body Mass Index Percentile, Food Addiction, and Addictive Phone Use. Appetite.

[52] Mann K.D., Howe L.D., Basterfield L., Parkinson K.N., Pearce M.S., Reilly J.K., Adamson A.J., Reilly J.J., Janssen X. (2017). Longitudinal Study of the Associations between Change in Sedentary Behavior and Change in Adiposity during Childhood and Adolescence: Gateshead Millennium Study. Int. J. Obes..

[53] Calcaterra V., Cena H., Rossi V., Santero S., Bianchi A., Zuccotti G. (2023). Ultra-Processed Food, Reward System and Childhood Obesity. Children.

[54] Sehn A.P., Silveira J.F.D.C., Brand C., Lemes V.B., Borfe L., Tornquist L., Pfeiffer K.A., Renner J.D.P., Andersen L.B., Burns R.D. (2024). Screen Time, Sleep Duration, Leisure Physical Activity, Obesity, and Cardiometabolic Risk in Children and Adolescents: A Cross-Lagged 2-Year Study. BMC Cardiovasc. Disord..

[55] Biddle S.J.H., García Bengoechea E., Wiesner G. (2017). Sedentary Behaviour and Adiposity in Youth: A Systematic Review of Reviews and Analysis of Causality. Int. J. Behav. Nutr. Phys. Act..

[56] Turconi G., Celsa M., Rezzani C., Biino G., Sartirana M.A., Roggi C. (2003). Reliability of a Dietary Questionnaire on Food Habits, Eating Behaviour and Nutritional Knowledge of Adolescents. Eur. J. Clin. Nutr..

[57] Maccarini B., Loperfido F., Bianco I., Sottotetti F., El Masri D., Ferrara C., Verme F., Cangelosi E., Meriggi N., De Filippo C. (2025). Offspring’s Exposome: A Narrative Review on the Influence of Early-Life Factors on Childhood Obesity Risk. Front. Nutr..

[58] Valerio G., Di Bonito P., Calcaterra V., Cherubini V., Corica D., De Sanctis L., Di Sessa A., Faienza M.F., Fornari E., Iughetti L. (2024). Cardiometabolic Risk in Children and Adolescents with Obesity: A Position Paper of the Italian Society for Pediatric Endocrinology and Diabetology. Ital. J. Pediatr..

[59] Jang E., Ko E., Sim J., Jeong M., Park S. (2024). *Mukbang* Media: Correlations with the Dietary Behavior of Children and Adolescents in Korea. Nutr. Res. Pract..

[60] Lundqvist M., Vogel N.E., Levin L.-Å. (2019). Effects of Eating Breakfast on Children and Adolescents: A Systematic Review of Potentially Relevant Outcomes in Economic Evaluations. Food Nutr. Res..

[61] Ricotti R., Caputo M., Monzani A., Pigni S., Antoniotti V., Bellone S., Prodam F. (2021). Breakfast Skipping, Weight, Cardiometabolic Risk, and Nutrition Quality in Children and Adolescents: A Systematic Review of Randomized Controlled and Intervention Longitudinal Trials. Nutrients.

[62] Szczudlik E., Stępniewska A., Bik-Multanowski M., Brandt-Heunemann S., Flehmig B., Małecka-Tendera E., Mazur A., Petriczko E., Ranke M.B., Wabitsch M. (2024). The Age of the Obesity Onset Is a Very Important Factor for the Development of Metabolic Complications and Cardiovascular Risk in Children and Adolescents with Severe Obesity. Eur. J. Pediatr..

[63] Khamzina M., Parab K.V., An R., Bullard T., Grigsby-Toussaint D.S. (2020). Impact of Pokémon Go on Physical Activity: A Systematic Review and Meta-Analysis. Am. J. Prev. Med..

[64] Vera-Ponce V.J., Ballena-Caicedo J., Zuzunaga-Montoya F.E., Gutierrez De Carrillo C.I. (2025). Effectiveness of Active Video Games for Promoting Physical Activity: An Umbrella Review. Front. Sports Act. Living.

[65] Wang J.-W., Zhu Z., Shuling Z., Fan J., Jin Y., Gao Z.-L., Chen W.-D., Li X. (2024). Effectiveness of mHealth App–Based Interventions for Increasing Physical Activity and Improving Physical Fitness in Children and Adolescents: Systematic Review and Meta-Analysis. JMIR mHealth uHealth.

[66] Conte G., Iorio G.D., Esposito D., Romano S., Panvino F., Maggi S., Altomonte B., Casini M.P., Ferrara M., Terrinoni A. (2025). Scrolling through Adolescence: A Systematic Review of the Impact of TikTok on Adolescent Mental Health. Eur. Child Adolesc. Psychiatry.

[67] Shakir R.N., Coates A.M., Olds T., Rowlands A., Tsiros M.D. (2018). Not All Sedentary Behaviour Is Equal: Children’s Adiposity and Sedentary Behaviour Volumes, Patterns and Types. Obes. Res. Clin. Pract..

[68] Mohd Saat N.Z., Hanawi S.A., Hanafiah H., Ahmad M., Farah N.M.F., Abdul Rahman N.A.A. (2024). Relationship of Screen Time with Anxiety, Depression, and Sleep Quality among Adolescents: A Cross-Sectional Study. Front. Public Health.

[69] Chaput J.-P., Willumsen J., Bull F., Chou R., Ekelund U., Firth J., Jago R., Ortega F.B., Katzmarzyk P.T. (2020). 2020 WHO Guidelines on Physical Activity and Sedentary Behaviour for Children and Adolescents Aged 5–17 Years: Summary of the Evidence. Int. J. Behav. Nutr. Phys. Act..

[70] (2020). WHO Guidelines on Physical Activity and Sedentary Behaviour.

[71] (2022). WHO Global Status Report on Physical Activity 2022.

[72] Orben A., Przybylski A.K., Blakemore S.-J., Kievit R.A. (2022). Windows of Developmental Sensitivity to Social Media. Nat. Commun..

[73] Saruco E., Pleger B. (2021). A Systematic Review of Obesity and Binge Eating Associated Impairment of the Cognitive Inhibition System. Front. Nutr..

[74] Blyth F., Haycraft E., Peral-Suarez A., Pearson N. (2025). Tracking and Changes in the Clustering of Physical Activity, Sedentary Behavior, Diet, and Sleep across Childhood and Adolescence: A Systematic Review. Obes. Rev..

[75] Wilhite K., Booker B., Huang B.-H., Antczak D., Corbett L., Parker P., Noetel M., Rissel C., Lonsdale C., Del Pozo Cruz B. (2023). Combinations of Physical Activity, Sedentary Behavior, and Sleep Duration and Their Associations with Physical, Psychological, and Educational Outcomes in Children and Adolescents: A Systematic Review. Am. J. Epidemiol..

[76] McHill A.W., Hull J.T., Klerman E.B. (2022). Chronic Circadian Disruption and Sleep Restriction Influence Subjective Hunger, Appetite, and Food Preference. Nutrients.

[77] Lee J.H., Cho J. (2022). Sleep and Obesity. Sleep Med. Clin..

[78] Klok M.D., Jakobsdottir S., Drent M.L. (2007). The Role of Leptin and Ghrelin in the Regulation of Food Intake and Body Weight in Humans: A Review. Obes. Rev..

[79] Gale E.L., Cecil J.E., Williams A.J. (2025). Shared Determinants of Poor Sleep, Obesity and Adiposity in Adolescents Aged 8–18-Years: A Systematic Review. J. Sleep Res..

[80] Abanoz E., Ulger Ozbek D., Güven Say A., Uzun Cicek A. (2025). Dysregulation of the Orexin–Leptin–Ghrelin Axis and Its Associations with Chronotype and Sleep Disturbances in Drug-Naïve Children with ADHD. Sleep Med..

[81] Taheri S., Lin L., Austin D., Young T., Mignot E. (2004). Short Sleep Duration Is Associated with Reduced Leptin, Elevated Ghrelin, and Increased Body Mass Index. PLoS Med..

[82] Lissak G. (2018). Adverse Physiological and Psychological Effects of Screen Time on Children and Adolescents: Literature Review and Case Study. Environ. Res..

[83] Stiglic N., Viner R.M. (2019). Effects of Screentime on the Health and Well-Being of Children and Adolescents: A Systematic Review of Reviews. BMJ Open.

[84] Liberali R., Del Castanhel F., Kupek E., Assis M.A.A.D. (2021). Latent Class Analysis of Lifestyle Risk Factors and Association with Overweight and/or Obesity in Children and Adolescents: Systematic Review. Child. Obes..

[85] Li C., Cheng G., Sha T., Cheng W., Yan Y. (2020). The Relationships between Screen Use and Health Indicators among Infants, Toddlers, and Preschoolers: A Meta-Analysis and Systematic Review. Int. J. Environ. Res. Public Health.

[86] Jiang Y., Hu J., Chen F., Liu B., Wei M., Xia W., Yan Y., Xie J., Du S., Tian X. (2025). Comprehensive Systematic Review and Meta-Analysis of Risk Factors for Childhood Obesity in China and Future Intervention Strategies. Lancet Reg. Health—West. Pac..

[87] Hu J., Xie J., Liu B., Peng W., Liu B., Yan Y., Zhang L., Wang X., Xi Y., Ma Y. (2026). Major Risk Factors of Obesity in China and Recommendations for Future Prevention and Control Efforts: A Systematic Review and Meta-Analysis. Lancet Reg. Health—West. Pac..

[88] Shibeshi A.H., Asfaw Z.G., Hasen A.A., Arge K.G., Hussen N.M., Seid A.A., Moloro A.H., Asebe H.A., Anbesu E.W., Asgedom D.K. (2026). Prevalence of Overweight and Obesity and Its Associated Factors among Preschool Children in Sub-Saharan Africa: A Systematic Review and Meta-Analysis. Adv. Nutr..

[89] Goodman W., Jackson S.E., McFerran E., Purves R., Redpath I., Beeken R.J. (2020). Association of Video Game Use with Body Mass Index and Other Energy-Balance Behaviors in Children. JAMA Pediatr..

[90] Nagata J.M., Iyer P., Chu J., Baker F.C., Gabriel K.P., Garber A.K., Murray S.B., Bibbins-Domingo K., Ganson K.T. (2021). Contemporary Screen Time Usage among Children 9–10-years-old Is Associated with Higher Body Mass Index Percentile at 1-year Follow-up: A Prospective Cohort Study. Pediatr. Obes..

[91] Miguel-Berges M.L., Mouratidou T., Santaliestra-Pasias A., Androutsos O., Iotova V., Galcheva S., De Craemer M., Cardon G., Koletzko B., Kulaga Z. (2023). Longitudinal Associations between Diet Quality, Sedentary Behaviours and Physical Activity and Risk of Overweight and Obesity in Preschool Children: The ToyBox-study. Pediatr. Obes..

[92] Carson V., Hunter S., Kuzik N., Gray C.E., Poitras V.J., Chaput J.-P., Saunders T.J., Katzmarzyk P.T., Okely A.D., Connor Gorber S. (2016). Systematic Review of Sedentary Behaviour and Health Indicators in School-Aged Children and Youth: An Update. Appl. Physiol. Nutr. Metab..

[93] LeBlanc A.G., Spence J.C., Carson V., Connor Gorber S., Dillman C., Janssen I., Kho M.E., Stearns J.A., Timmons B.W., Tremblay M.S. (2012). Systematic Review of Sedentary Behaviour and Health Indicators in the Early Years (Aged 0–4 Years). Appl. Physiol. Nutr. Metab..

[94] Tremblay M.S., LeBlanc A.G., Kho M.E., Saunders T.J., Larouche R., Colley R.C., Goldfield G., Gorber S. (2011). Systematic Review of Sedentary Behaviour and Health Indicators in School-Aged Children and Youth. Int. J. Behav. Nutr. Phys. Act..

[95] Fang K., Mu M., Liu K., He Y. (2019). Screen Time and Childhood Overweight/Obesity: A Systematic Review and Meta-analysis. Child.

[96] Nagata J.M., Smith N., Alsamman S., Lee C.M., Dooley E.E., Kiss O., Ganson K.T., Wing D., Baker F.C., Gabriel K.P. (2023). Association of Physical Activity and Screen Time with Body Mass Index Among US Adolescents. JAMA Netw. Open.

[97] Nightingale C.M., Rudnicka A.R., Donin A.S., Sattar N., Cook D.G., Whincup P.H., Owen C.G. (2017). Screen Time Is Associated with Adiposity and Insulin Resistance in Children. Arch. Dis. Child..

[98] Mihrshahi S., Drayton B.A., Bauman A.E., Hardy L.L. (2017). Associations between Childhood Overweight, Obesity, Abdominal Obesity and Obesogenic Behaviors and Practices in Australian Homes. BMC Public Health.

[99] Chahal H., Fung C., Kuhle S., Veugelers P.J. (2013). Availability and Night-time Use of Electronic Entertainment and Communication Devices Are Associated with Short Sleep Duration and Obesity among C Anadian Children. Pediatr. Obes..

[100] Staiano A.E., Harrington D.M., Broyles S.T., Gupta A.K., Katzmarzyk P.T. (2013). Television, Adiposity, and Cardiometabolic Risk in Children and Adolescents. Am. J. Prev. Med..

[101] Neshteruk C.D., Tripicchio G.L., Lobaugh S., Vaughn A.E., Luecking C.T., Mazzucca S., Ward D.S. (2021). Screen Time Parenting Practices and Associations with Preschool Children’s TV Viewing and Weight-Related Outcomes. Int. J. Environ. Res. Public Health.

[102] Marker C., Gnambs T., Appel M. (2022). Exploring the Myth of the Chubby Gamer: A Meta-Analysis on Sedentary Video Gaming and Body Mass. Soc. Sci. Med..

[103] Wachira L.-J.M., Muthuri S.K., Ochola S.A., Onywera V.O., Tremblay M.S. (2018). Screen-Based Sedentary Behaviour and Adiposity among School Children: Results from International Study of Childhood Obesity, Lifestyle and the Environment (ISCOLE)—Kenya. PLoS ONE.

[104] Ghasemirad M., Ketabi L., Fayyazishishavan E., Hojati A., Maleki Z.H., Gerami M.H., Moradzadeh M., Fernandez J.H.O., Akhavan-Sigari R. (2023). The Association between Screen Use and Central Obesity among Children and Adolescents: A Systematic Review and Meta-Analysis. J. Health Popul. Nutr..

[105] Mark A.E., Janssen I. (2008). Relationship between Screen Time and Metabolic Syndrome in Adolescents. J. Public Health.

[106] Goldfield G.S., Kenny G.P., Hadjiyannakis S., Phillips P., Alberga A.S., Saunders T.J., Tremblay M.S., Malcolm J., Prud’homme D., Gougeon R. (2011). Video Game Playing Is Independently Associated with Blood Pressure and Lipids in Overweight and Obese Adolescents. PLoS ONE.

[107] Martinez-Gomez D., Rey-López J.P., Chillón P., Gómez-Martínez S., Vicente-Rodríguez G., Martín-Matillas M., Garcia-Fuentes M., Delgado M., Moreno L.A., Veiga O.L. (2010). Excessive TV Viewing and Cardiovascular Disease Risk Factors in Adolescents. The AVENA Cross-Sectional Study. BMC Public Health.

[108] Musa S., Elyamani R., Dergaa I. (2022). COVID-19 and Screen-Based Sedentary Behaviour: Systematic Review of Digital Screen Time and Metabolic Syndrome in Adolescents. PLoS ONE.

[109] Horner D., Jahn M., Bønnelykke K., Chawes B., Flensborg-Madsen T., Schoos A.-M.M., Stokholm J., Rasmussen M.A. (2025). Screen Time Is Associated with Cardiometabolic and Cardiovascular Disease Risk in Childhood and Adolescence. J. Am. Heart Assoc..

[110] Kunin-Batson A.S., Crain A.L., Sherwood N.E., Kelly A.S., Kharbanda E.O., Gunnar M.R., Haapala J., Seburg E.M., French S.A. (2024). Do Children’s Health Behaviors Buffer the Impact of Cumulative Environmental Stress on Emerging Cardiometabolic Risk?. J. Am. Heart Assoc..

[111] Lee H.-S., Jeong W.-W., Choi Y.-J., Seo Y.-G., Noh H.-M., Song H.-J., Paek Y.-J., Kim Y.-M., Lim H.-J., Lee H.-J. (2019). Association between Physical Fitness and Cardiometabolic Risk of Children and Adolescents in Korea. Korean J. Fam. Med..

[112] Saunders T.J., Tremblay M.S., Mathieu M.-È., Henderson M., O’Loughlin J., Tremblay A., Chaput J.-P., QUALITY Cohort Research Group (2013). Associations of Sedentary Behavior, Sedentary Bouts and Breaks in Sedentary Time with Cardiometabolic Risk in Children with a Family History of Obesity. PLoS ONE.

[113] Sehn A.P., Brand C., de Castro Silveira J.F., Andersen L.B., Gaya A.R., Todendi P.F., de Moura Valim A.R., Reuter C.P. (2022). What Is the Role of Cardiorespiratory Fitness and Sedentary Behavior in Relationship between the Genetic Predisposition to Obesity and Cardiometabolic Risk Score?. BMC Cardiovasc. Disord..

[114] Hardy L.L., Denney-Wilson E., Thrift A.P., Okely A.D., Baur L.A. (2010). Screen Time and Metabolic Risk Factors Among Adolescents. Arch. Pediatr. Adolesc. Med..

[115] Henderson M., Benedetti A., Barnett T.A., Mathieu M.-E., Deladoëy J., Gray-Donald K. (2016). Influence of Adiposity, Physical Activity, Fitness, and Screen Time on Insulin Dynamics over 2 Years in Children. JAMA Pediatr..

[116] van Ekris E., Altenburg T.M., Singh A.S., Proper K.I., Heymans M.W., Chinapaw M.J.M. (2016). An Evidence-Update on the Prospective Relationship between Childhood Sedentary Behaviour and Biomedical Health Indicators: A Systematic Review and Meta-Analysis. Obes. Rev..

[117] Vanderloo L.M., Keown-Stoneman C.D.G., Sivanesan H., Parkin P.C., Maguire J.L., Anderson L.N., Tremblay M.S., Birken C.S., TARGet Kids! Collaborative (2020). Association of Screen Time and Cardiometabolic Risk in School-Aged Children. Prev. Med. Rep..

[118] Sivanesan H., Vanderloo L.M., Keown-Stoneman C.D.G., Parkin P.C., Maguire J.L., Birken C.S., TARGet Kids! Collaboration (2020). The Association between Screen Time and Cardiometabolic Risk in Young Children. Int. J. Behav. Nutr. Phys. Act..

[119] Berentzen N.E., Smit H.A., van Rossem L., Gehring U., Kerkhof M., Postma D.S., Boshuizen H.C., Wijga A.H. (2014). Screen Time, Adiposity and Cardiometabolic Markers: Mediation by Physical Activity, Not Snacking, among 11-Year-Old Children. Int. J. Obes..

[120] Pardee P.E., Norman G.J., Lustig R.H., Preud’homme D., Schwimmer J.B. (2007). Television Viewing and Hypertension in Obese Children. Am. J. Prev. Med..

[121] Martinez-Gomez D., Tucker J., Heelan K.A., Welk G.J., Eisenmann J.C. (2009). Associations Between Sedentary Behavior and Blood Pressure in Young Children. Arch. Pediatr. Adolesc. Med..

[122] Cassidy-Bushrow A.E., Johnson D.A., Peters R.M., Burmeister C., Joseph C.L.M. (2015). Time Spent on the Internet and Adolescent Blood Pressure. J. Sch. Nurs..

[123] Gopinath B., Hardy L.L., Kifley A., Baur L.A., Mitchell P. (2014). Activity Behaviors in Schoolchildren and Subsequent 5-Yr Change in Blood Pressure. Med. Sci. Sports Exerc..

[124] Aljahdali A.A., Baylin A., Ruiz-Narvaez E.A., Kim H.M., Cantoral A., Tellez-Rojo M.M., Banker M., Peterson K.E. (2022). Sedentary Patterns and Cardiometabolic Risk Factors in Mexican Children and Adolescents: Analysis of Longitudinal Data. Int. J. Behav. Nutr. Phys. Act..

[125] Tornquist D., Tornquist L., Sehn A.P., Schneiders L.d.B., Pollo Renner J.D., Rech Franke S.I., Reuter C.P., Kelishadi R. (2022). Cardiorespiratory Fitness, Screen Time and Cardiometabolic Risk in South Brazilian School Children. Ann. Hum. Biol..

[126] Bhor S., Bhalerao A.A., Shah N., Khadilkar V., Khadilkar A.V. (2026). Screen Time and Its Impact on Cardiometabolic Risk Factors among Children and Youth with Type 1 Diabetes (T1D). J. Pharm. Bioallied Sci..

[127] Domingues-Montanari S. (2017). Clinical and Psychological Effects of Excessive Screen Time on Children. J. Paediatr. Child Health.

[128] Santos R.M.S., Mendes C.G., Sen Bressani G.Y., De Alcantara Ventura S., De Almeida Nogueira Y.J., De Miranda D.M., Romano-Silva M.A. (2023). The Associations between Screen Time and Mental Health in Adolescents: A Systematic Review. BMC Psychol..

[129] Reichenberger J., Schnepper R., Arend A.-K., Blechert J. (2020). Emotional Eating in Healthy Individuals and Patients with an Eating Disorder: Evidence from Psychometric, Experimental and Naturalistic Studies. Proc. Nutr. Soc..

[130] Keeler J., Conde Ludtke L., Yang Q., Raschke Rameh V., Ward R., Treasure J., Carter B. (2026). Associations of Problematic Smartphone Use and Smartphone Screen Time with Eating Disorder Psychopathology in Non-Clinical Samples: A Systematic Review. JMIR Ment. Health.

[131] Cerolini S., Nowicki G.P., Rodgers R.F. (2025). The Interplay between Social Media Use, Poor Sleep, and Disordered Eating: A Narrative Review. Cogent Ment. Health.

[132] Zhou J., Chen Y., Ji S., Qu J., Bu Y., Li W., Zhou Z., Wang X., Fu X., Liu Y. (2024). Sleep Quality and Emotional Eating in College Students: A Moderated Mediation Model of Depression and Physical Activity Levels. J. Eat. Disord..

[133] Brown T., Tanti V., Wilson N., Griffiths M.D., Lubman D., Hein K., Stavropoulos V. (2026). Digital Engagement Profiles and Binge Eating Symptoms in Adolescents: A Person-Centred, Longitudinal Analysis. Compr. Psychiatry.

[134] Al-Shoaibi A.A.A., Shao I.Y., Ganson K.T., Lavender J.M., Testa A., Kiss O., He J., Glidden D.V., Baker F.C., Nagata J.M. (2024). Prospective Association of Screen Time with Binge-eating Disorder among Adolescents in the United States: The Mediating Role of Depression. Int. J. Eat. Disord..

[135] Zheng Q., Chen M., Hu J., Zhou T., Wang P. (2024). Appearance Comparison, Body Appreciation, and Adolescent Depressive Symptoms: Roles of Gender, Age, and Body-Mass Index. Psychol. Res. Behav. Manag..

[136] Vuong A.T., Jarman H.K., Doley J.R., McLean S.A. (2021). Social Media Use and Body Dissatisfaction in Adolescents: The Moderating Role of Thin- and Muscular-Ideal Internalisation. Int. J. Environ. Res. Public Health.

[137] Merino M., Tornero-Aguilera J.F., Rubio-Zarapuz A., Villanueva-Tobaldo C.V., Martín-Rodríguez A., Clemente-Suárez V.J. (2024). Body Perceptions and Psychological Well-Being: A Review of the Impact of Social Media and Physical Measurements on Self-Esteem and Mental Health with a Focus on Body Image Satisfaction and Its Relationship with Cultural and Gender Factors. Healthcare.

[138] Surprenant R., Bezeau D., Cabot I., Smith J., Kim H.S., Fitzpatrick C. (2026). Physical Activity Moderates the Relationship Between Screen Time and Body Dissatisfaction in Early Adulthood. Health Educ. Behav..

[139] Lo Coco G., Rodgers R., Harris E.A., Markey C., Sicilia A., Aimé A., Dion J., Salerno L., Hayami-Chisuwa N., White H.J. (2026). Investigating the Relation between Social Media, Dating App Use and Body Image Dimensions: A Cross-country Study. Br. J. Health Psychol..

[140] Tang L., Rifas-Shiman S.L., Field A.E., Austin S.B., Haines J. (2022). Self-Reported Total Screen Time and Viewing Modes Are Associated with Body Dissatisfaction, Disordered Eating, and Cosmetic Surgery Intentions among Young Adults. Nutrients.

[141] Basilico S., Conti M.V., Ardoino I., Breda C., Loperfido F., Orsini F., Fernandez M.L.O., Pierini L., Bonizzoni S.C., Modena E. (2025). Lights and Shadows of a Primary School-Based Nutrition Education Program in Italy: Insights from the LIVELY Project. Nutrients.

[142] Conti M.V., Vincenti A., Beretta A., Calcaterra V., Taranto S., Diotti M., Zuccotti G., Cena H. (2024). Planetary Health Diet for Childhood Obesity Prevention: Integrating Nutritional Health with Environmental Stewardship. Nutrients.

[143] Breda C., Santero S., Conti M.V., Cena H. (2025). Programmes to Manage Food Selectivity in Individuals with Autism Spectrum Disorder. Nutr. Res. Rev..

[144] De Giuseppe R., Di Napoli I., Porri D., Cena H. (2019). Pediatric Obesity and Eating Disorders Symptoms: The Role of the Multidisciplinary Treatment. A Systematic Review. Front. Pediatr..

