# Supplementary material for: Screen Time, Unhealthy Eating Behaviors, and Associated Health Risks in Children: A Narrative Review
Source: Children (Basel). 2026 Jun 30;13(7):887. doi: 10.3390/children13070887 (PMC13406753; doi:10.3390/children13070887)
Supplement: Supplementary file 1 [file children-13-00887-s001.zip › children-4353513-supplementary.pdf]

Table S1. Search strategy used across databases.

Database	Search strategy
**PubMed**	("screen time" OR "screen exposure" OR "digital media" OR television OR smartphone OR tablet OR videogame* OR "social media" OR "digital marketing") AND (child* OR adolescen* OR pediatric OR preschool OR youth) AND ("eating behavior" OR "dietary habits" OR "food intake" OR snacking OR "breakfast skipping" OR "ultra-processed food" OR "food advertising" OR "food marketing") AND (obesity OR adiposity OR "body mass index" OR cardiometabolic OR "insulin resistance" OR dyslipidemia OR hypertension OR sleep OR depression OR anxiety OR "emotional eating" OR "binge eating" OR "body image")
**Scopus**	TITLE-ABS-KEY ("screen time" OR "screen exposure" OR "digital media" OR television OR smartphone OR tablet OR videogame* OR "social media" OR "digital marketing") AND TITLE-ABS-KEY (child* OR adolescen* OR pediatric OR preschool OR youth) AND TITLE-ABS-KEY ("eating behavior" OR "dietary habits" OR "food intake" OR snacking OR "breakfast skipping" OR "ultra-processed food" OR "food advertising" OR "food marketing") AND TITLE-ABS-KEY (obesity OR adiposity OR "body mass index" OR cardiometabolic OR "insulin resistance" OR dyslipidemia OR hypertension OR sleep OR depression OR anxiety OR "emotional eating" OR "binge eating" OR "body image")
**Web of Science**	TS=("screen time" OR "screen exposure" OR "digital media" OR television OR smartphone OR tablet OR videogame* OR "social media" OR "digital marketing") AND TS=(child* OR adolescen* OR pediatric OR preschool OR youth) AND TS=("eating behavior" OR "dietary habits" OR "food intake" OR snacking OR "breakfast skipping" OR "ultra-processed food" OR "food advertising" OR "food marketing") AND TS=(obesity OR adiposity OR "body mass index" OR cardiometabolic OR "insulin resistance" OR dyslipidemia OR hypertension OR sleep OR depression OR anxiety OR "emotional eating" OR "binge eating" OR "body image")
